# Descriptions of Four New Species in Cunninghamellaceae (Mucoromycota) from the Brazilian Savanna Through Integrative Taxonomy

**DOI:** 10.3390/jof12050329

**Published:** 2026-05-02

**Authors:** Leslie Waren Silva de Freitas, Layanne de Oliveira Ferro, Andre Rodrigues, Camila Santana de Oliveira, Mateus Oliveira da Cruz, Jadson Diogo Pereira Bezerra, Hyang Burm Lee, Cristina Maria de Souza-Motta, Maria Alice Barbosa dos Santos, Roger Fagner Ribeiro Melo, André Luiz Cabral Monteiro de Azevedo Santiago

**Affiliations:** 1 Departamento de Mycologia, Universidade Federal de Pernambuco, Avenida da Engenharia, s/n, Recife 50740-600, Pernambuco, Brazil; lesliewaren@gmail.com (L.W.S.d.F.); cristina.motta@ufpe.br (C.M.d.S.-M.); alice.barbosasantos@ufpe.br (M.A.B.d.S.); roger.melo@ufpe.br (R.F.R.M.); andrelcabral@msn.com (A.L.C.M.d.A.S.); 2 Laboratório de Micologia, Instituto de Patologia Tropical e Saúde Pública (IPTSP), Universidade Federal de Goiás, Rua 235, s/n, Goiânia 74605-050, Goiás, Brazil; layanne.ferro93@gmail.com (L.d.O.F.); santana.oliveira@discente.ufg.br (C.S.d.O.); 3 Department of General and Applied Biology, São Paulo State University (UNESP), Av. 24-A, 1515, Bela Vista, Rio Claro 13506-900, São Paulo, Brazil; cruzmofungi@gmail.com; 4 Environmental Microbiology Laboratory, Department of Agricultural Biological Chemistry, College of Agriculture and Life Sciences, Chonnam National University, Yongbong-Dong 300, Buk-Gu, Gwangju 61186, Republic of Korea

**Keywords:** four new taxa, *Absidia*, *Gongronella*, Cerrado biome, soil, taxonomy

## Abstract

During a survey on *Mucorales* fungi from soil in the world’s most biodiverse savanna, the the Brazilian Cerrado, nine specimens belonging to the *Cunninghamellace* were isolated. Morphological, multiloci analyses (ITS-nLSU-*act*) and maximum temperature growth data revealed that those specimens represent four new species: two in *Absidia* and two in *Gongronella*. Morphological characteristics of the isolates distinguishes them from other species: *Absidia rhizoidea* sp. nov. forms rhizopodiform rhizoids at the end of stolons, commonly next to the sporangiophores; *A. variabilis* sp. nov., mostly with slightly dorsiventrally flattened sporangia; *Gongronella longapophysata* sp. nov., which forms a long apophysis below sporangia; and *G. verticilatta* sp. nov., with whorled-branched sporangiophores. The maximum temperatures growth (Tmax) of those new species are as follows: *A. rhizoidea* (33 °C on MEA and 32 °C on PDA), *A. variabilis* (31 °C on MEA and 32 °C on PDA), *G. longapophysata* (32 °C on MEA and 33 °C on PDA), and *G. verticilatta* (31 °C on MEA and PDA). The present study highlights and discusses the micromorphological, physiological (Tmax) and phylogenetic characteristics of the new species.

## 1. Introduction

The Cerrado biome (Brazilian Savanna), located in the Central Plateau of Brazil, is the second largest biome in extension in the country (over 2,000,000 km^2^, covering about 23% of the Brazilian territory), only surpassed by the Amazon Rainforest [1,2]. Different types of forest formations are observed in the Cerrado, such as riparian forest, gallery forest, dry forest, “cerradão”, savanna and grassland [1,3]. To date (as of 3 March 2026), 613 fungal species have been reported from the Brazilian Cerrado, which corresponds to 0.43% of circa (ca.) 140,000 known fungal species [4] and to 7.74% of the 7918 fungal species registered in Brazil [5]. Regarding the order *Mucorales* Dumort., only 17 species have been officially registered in this biome [5], from which *Backusella gigacellularis* J.I. de Souza, Pires-Zottarelli & Harakava [6]; and *Isomucor trufemiae* J.I. de Souza, Pires-Zottarelli & Harakava [7] were introduced as new species. As for *Cunninghamellaceae* Naumov ex R.K. Benj., reports of species in this family in the Cerrado are restricted to five species, namely, *Absidia caerulea* Bainier, *A. cylindrospora* Hagem, *A. spinosa* Lendn., *Cunninghamella elegans* Lendn., and *C. phaeospora* Boedijn [5]. Despite being one of the largest Brazilian biomes, our knowledge about fungal diversity of the Cerrado is limited, especially regarding *Cunninghamellaceae*. Furthermore, this biome suffers from constant deforestation, soil degradation, water depletion and fires [8], which makes its unknown mycodiversity even more threatened, making its discovery urgent.

Species in the *Cunninghamellaceae* form sporangiophores with a columellate sporangium or sporophores with a terminal vesicle with uni- to multispored pedicellate sporangiola. Zygospores are globose with opposed suspensors [9,10]. Naumov [11] accepted *Cunninghamella* Matr., *Sigmoideomyces* Thaxt., and *Thamnocephalis* Blakeslee in this family, and other genera were added to *Cunninghamellaceae* later, such as *Chaetocladium* Fresen. [12,13], *Mycotypha* Fenner [14,15] and *Phascolomyces* Boedijn [16]. However, Benny et al. [17] and Voigt and Wöstemeyer [18] considered the family as monogeneric, with only *Cunninghamella.* On the other hand, Voigt [10] and Wijayawardene et al. [4] accepted *Absidia* Tiegh., *Chlamydoabsidia* Hesselt. & J. J. Ellis, *Cunninghamella*, *Gongronella* Ribaldi, *Halteromyces* Shipton & Schipper, and *Hesseltinella* H.P. Upadhyay in the *Cunninghamellaceae*. Here we are following the classification of Wijayawardene et al. [4].

*Absidia* species are commonly isolated from herbivore dung, soil, stored food and plant remains [19]. They form stolons and rhizoids not opposed to the sporangium [20,21]. The unbranched or branched sporangiophores arise isolated or in whorls from the stolon. Sporangia are apophysate, globose, subglobose and pyriform (when considering the apophysis), and the varied-shaped columellae commonly present varied-shaped projections on their surface. Zygosporangia are globose to subglobose and born from opposed suspensors with appendages [22]. Some species of this genus are biotechnologically relevant, such as *A. caerulea* Bainier and *A. glauca* Hagem, which can produce chitosan and chitin deacetylase [23] and biotransform flavonoids (chrysin, apigenin, luteolin, and diosmetin) and flavanones (pinocembrin, naringenin, eriodictyol, and hesperetin) [24]. *Absidia fusca* Linnem. and *A. cylindrospora* Hagem can degrade polycyclic aromatic hydrocarbon compounds [25]. Furthermore, experiments carried out with *A. cylindrospora* highlighted its capacity to biosorb heavy metals such as cadmium, copper and lead [26]. The genus currently comprises 94 species [27].

Most of the *Gongronella* species have been reported from soil samples, with only *G. namwonensis* Hyang B. Lee, A.L. Santiago & H.J. Lim being isolated from fresh water [28]. Species of this genus show morphological characteristics similar to the ones of *Absidia* species, such as the formation of stolons and rhizoids (although discrete) not opposed to the sporangium and sporangiophores with apophysate sporangia. The columellae are commonly globose, hemispherical and subglobose, and sterile sporangia are formed in some species [9,21]. Sporangiospores are commonly reniform, but may also be lacrimoid, globose and subglobose, and zygosporangia are born from opposed suspensors, without appendages [21,29,30]. *Gongronella butleri* (Lendn.) Peyronel & Dal Vesco can produce pectinases [31], chitin deacetylase [32], amylases [33], beta-glucosidases [34], and chitosan [35]. This genus currently comprises 28 species [27].

During a survey of *Mucorales* fungi from the soils of the Chapada das Mesas, an area of Cerrado located in the state of Maranhão, Northeastern Brazil, we discovered four specimens of *Gongronella* and five of *Absidia* (*Cunninghamellaceae*) that differ morphologically and phylogenetically (ITS-LSU-*act*) from other species. This paper proposes two new species of *Absidia* and two of *Gongronella*, as well as discusses their morphology and phylogeny.

## 2. Materials and Methods

### 2.1. Sampling Site and Soil Collection

Soil samples were collected in the Chapada das Mesas region (7°17′51.2″ S 47°28′12.2″ W), which has circa (ca.) 160,000 ha and is located within the municipalities of Carolina, Estreito, and Riachão, in the middle of the Tocantins River basin. The vegetation cover of the Chapada das Mesas is typical of the Cerrado biome with formations of gallery forest; riparian forest; dry forest; herbaceous-shrub physiognomy with spaced shrubs and subshrubs, known as “campo sujo”; and herbaceous physiognomy with few shrubs and no trees, known as “campo limpo” [36,37]. The local soil types are cambisols, red-yellow latosols, quartz sands, and the climate is humid and tropical, with temperatures ranging from 26 to 36 °C [37].

Soil collection was carried out randomly in three different areas near the Parque Nacional da Chapada das Mesas: Cachoeira da Mansinha (7°07′54.0″ S 47°26′55.1″ W), Cachoeira do Dodô (7°05′33.2″ S 47°26′35.5″ W), Encanto Azul (7°14′00.6″ S 46°24′24.3″ W) and São Romão (6°02′09.1″ S 46°34′03.5″ W). The soil samples were collected at 5 cm deep, stored in plastic bags and kept in styrofoam boxes with ice during transportation to the Laboratório de Fungos Zigospóricos of the Universidade Federal de Pernambuco, UFPE.

### 2.2. Isolation, Purification and Deposit

Five milligrams of soil were added to Petri dishes with wheat germ agar medium [38] with chloramphenicol (80 mg L^−1^), in triplicate. Colony growth was monitored for 96 h at room temperature (28 ± 2 °C). Fragments of the colonies were transferred to Petri dishes with potato dextrose agar (PDA; HIMEDIA, Vadhani, India) [38] with chloramphenicol (80 mg L^−1^). The lyophilized holotypes and living cultures were deposited at the URM culture collection of the Universidade Federal de Pernambuco (Recife, Brazil).

### 2.3. Growth Experiments and Micromorphology

Strains were grown in triplicate on potato dextrose agar (PDA) and malt extract agar (MEA) [39], and incubated at 10, 15, 20, 25, 30, and 35 °C. Colony growth was monitored for 8 days, with colony diameters measured every 24 h. Growth results are shown as the arithmetic mean for all isolates of each species on both PDA and MEA. The maximum growth temperature (Tmax) was determined by incubating all the strains on MEA and PDA at temperatures one degree higher than the last temperature with growth. The Tmax of 50 *Absidia* species retrieved from literature plus four new species (two of *Absidia* and two of *Gongronella*) accessed in this work is shown in Table 1.

For observation, mycelial fragments were removed from the cultures, mounted on microscope slides with 3% KOH and/or Amman blue, and observed under a light microscope Leica DM 500 (Leica Microsystems, Wetzlar, Germany). Images were captured using the Leica DM 2500 microscope (Leica Microsystems, Wetzlar, Germany) equipped with a Leica flexacam C3 and processed using the Leica Application Suite X 3.8.1.2 software. One hundred measurements were made for each fungal structure. Measurements of the structures considered the length × width range, including outliers in parentheses. The colony color designations were determined according to Kornerup and Wanscher [40].

**Table 1:** Maximum growth temperatures of 52 *Absidia* and two *Gongronella* species. New species described in this study are in bold.

Species	Country	Tmax (°C)		
		MEA	PDA	SMA
*Absidia abundans* [41]	China	31	-	-
*Absidia ampullaceae* [42]	China	30	-	-
*Absidia arrhiza* [43]	China	-	33	-
*Absidia biappendiculata* [42]	China	35	-	-
*Absidia brunnea* [42]	China	35	-	-
*Absidia cheongyangensis* [20]	Korea	33	33	33
*Absidia chinensis* [42]	China	30	-	-
*Absidia cinerea* [42]	China	35	-	-
*Absidia collariata* [44]	China	-	29	-
*Absidia crystalloides* [45]	China	-	32	-
*Absidia digitula* [42]	China	32	-	-
*Absidia fluvii* [20]	Korea	32	32	32
*Absidia frigida* [46]	China	24	-	-
*Absidia gemella* [46]	China	29	-	-
*Absidia globospora* [47]	China	28	28	-
*Absidia hainanensis* [44]	China	-	34	-
*Absidia healeya* [48]	Australia	-	30	-
*Absidia jiangxiensis* [42]	China	31	-	-
*Absidia kunryangriensis* [20]	Korea	31	32	-
*Absidia lobata* [41]	China	26	-	-
*Absidia longissima* [46]	China	36	-	-
*Absidia medula* [47]	China	32	32	-
*Absidia menglianensis* [49]	China	-	36	-
*Absidia nigra* [42]	China	31	-	-
*Absidia oblongispora* [42]	China	32	-	-
*Absidia ovalispora* [50]	China	-	-	37
*Absidia pacifica* [45]	China	-	35	-
*Absidia paracylindrospora* [20]	Korea	31	32	31
*Absidia pararepens* [20]	Korea	32	32	32
*Absidia pateriformis* [45]	China	-	30	-
*Absidia purpurea* [42]	China	30	-	-
*Absidia pyriformis* [44]	China	-	33	-
*Absidia radiata* [41]	China	32	-	-
***Absidia rhizoidea***	**Brazil**	**33**	**31**	**-**
*Absidia sphaerica* [43]	China	-	33	-
*Absidia sichuanensis* [41]	China	28	-	-
*Absidia simplex* [43]	China	-	29	-
*Absidia sympodialis* [42]	China	33	-	-
*Absidia tarda* [20]	Brazil	36	36	36
*Absidia tardiva* [44]	China	-	27	-
*Absidia terrestris* [51]	Mexico	27	-	-
*Absidia thailandica* [52]	Thailand	-	27	-
*Absidia tibetensis* [44]	China	-	30	-
*Absidia turgida* [47]	China	32	32	-
***Absidia variabilis***	**Brazil**	**31**	**32**	**-**
*Absidia varians* [20]	China	29	-	-
*Absidia variiprojecta* [20]	Brazil	32	32	33
*Absidia variispora* [20]	Brazil	32	32	32
*Absidia virescens* [42]	China	33	-	-
*Absidia viridis* [43]	China	-	29	-
*Absidia yunnanensis* [41]	China	32	-	-
*Absidia zonata* [47]	China	37	37	-
***Gongronella longapophysata***	**Brazil**	**32**	**33**	**-**
***G. verticilatta***	**Brazil**	**31**	**31**	**-**

Legend: PDA—potato dextrose agar; MEA—malt extract agar; SMA—synthetic *Mucor* agar.

### 2.4. DNA Extraction, PCR Amplification and Sequencing

Genomic DNA extraction was carried out with the Wizard Genomic DNA Purification Kit (Promega), following the manufacturer’s guidelines. PCR amplifications for the internal transcribed spacer (ITS), part of the nuclear ribosomal large subunit (nLSU), and actin (*act*) were performed using primers and PCR conditions, as listed in Table 2. The amplification protocol was performed as described by de Freitas et al. [21,53]. Subsequently, bidirectional sequencing was performed with the same primers using the BigDye^®^ Terminator v.3.1 Cycle Sequencing Kit (Applied Biosystems Life Technologies, Carlsbad, CA, USA) at the Laboratório de Ecologia e Sistemática de Fungos—LESF, UNESP, Rio Claro, São Paulo and at the Centro Multiusuário de Pesquisa de Bioinsumos e Tecnologias em Saúde (CMBiotecs, IPTSP, UFG), Goiânia, Goiás. The sequences obtained in this study are available in the NCBI GenBank database (Table 3).

### 2.5. Phylogenetic Analysis

Phylogenetic analyses were performed using sequences generated in this study and reference sequences obtained from the GenBank database, based on markermultiloci concatenated analysis (ITS, LSU, and *act*). The sequence datasets used in this study were constructed based on studies on *Absidia* [20,49] and *Gongronella* [21,30]. The sequences were aligned using the MAFFT v7 online interface [56,57] and manually edited using MEGA v7 software [58]. The loci were concatenated using Mesquite v3.61 software [59].

The data were first analyzed based on Maximum Likelihood (ML) analysis using IQ-TREE v1.6.12 software [60] and RAxML HPC BlackBox v8.2.12 [61]. The RAxML-HPC BlackBox ML analysis was performed using the default system options in the CIPRES Science Gateway [62] with 5000 bootstrap replicates. The IQ-TREE ML analysis involved 5000 replications, and the ultrafast bootstrap (UFboot2) method was used to calculate the branch support [63]. The ModelFinder software (http://iqtree.cibiv.univie.ac.at, accessed on 3 March 2016) included in IQ-TREE v. 1.6.12 [64] was used to determine the partitioning strategy and models based on the Akaike information criterion. The combined datasets were also analyzed based on Bayesian inference (BI) conducted using MrBayes v3.2.7a [65] in the CIPRES Science Gateway, using the same models and partitions as in the ML analysis (IQ-TREE). The BI analysis was conducted with 5 × 10^7^ generations and a burning value of 25%, with chains sampled every 1000 generations. The resulting phylogenetic trees were visualized using FigTree v1.4.4 [66]. Values ≥ 0.95 BI posterior probability (BPP) and bootstrap support (BS) from IQ-TREE UFboot2-BS and RAxML-BS analyses (both BS ≥ 70%) were plotted near the nodes. The final combined alignment was deposited in Figshare (Study ID 10.6084/m9.figshare.31438165).

## 3. Results

### 3.1. Phylogenetic Analyses

#### 3.1.1. *Absidia*

**Figure 1:**
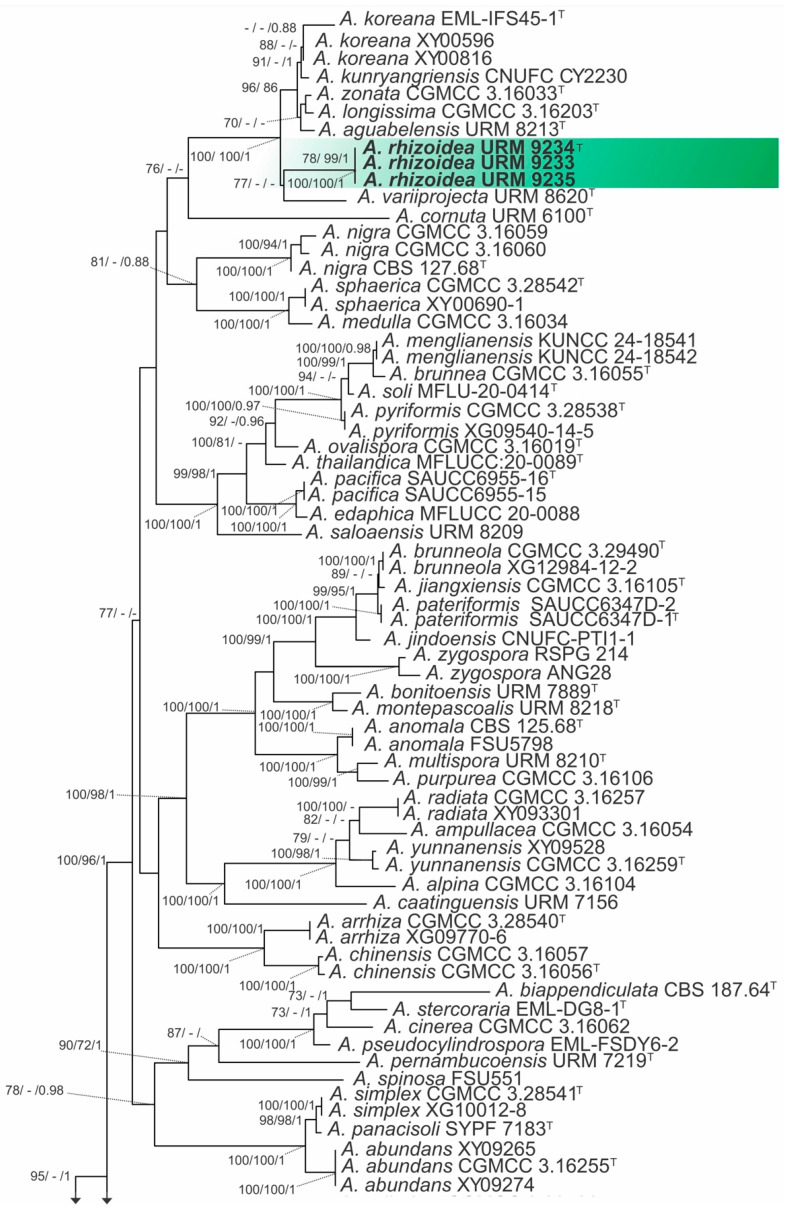

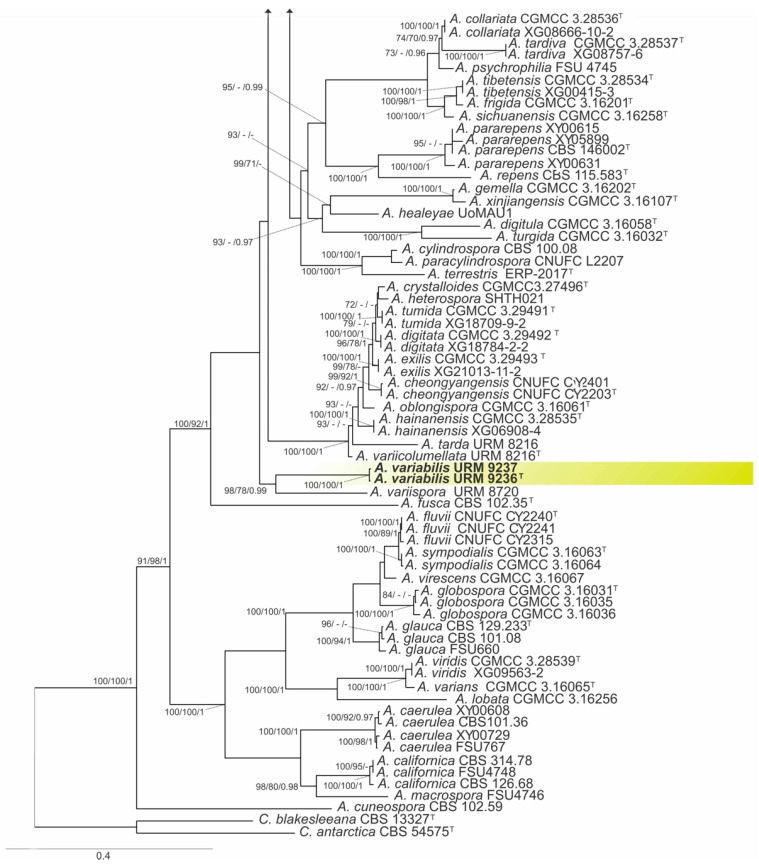
Maximum Likelihood (ML) phylogenetic tree of the combined ITS, LSU and *act* sequences of *Absidia* species. The specimens obtained in this study are in bold and highlighted in yellow or green Ex-type strains are marked with (T). Confidence values for ML-BS ≥ 70% (UFboot2/RAxML) and BPP ≥ 0.95 are included near the nodes and “-” indicates statistical support below the threshold values. The tree was rooted to *Cunninghamella blakesleeana* (CBS 133.27) and *Cunninghamella antarctica* (CBS 545.75).

The combined matrix of three markers of *Absidia* species (ITS, LSU, and *act*) comprised sequences from 135 strains, including the outgroup *Cunninghamella blakesleeana* (CBS 133.27) and *Cunninghamella antarctica* (CBS 545.75), with a total of 2940 characters, including gaps, with 1267 characters for ITS, 823 for LSU, and 850 for *act*. The models used for IQ-TREE ML and BI were HKY+I+G for ITS, GTR+I+G for LSU and GTR+G for *act*. For the ML analysis (RAxML-HPC BlackBox) the model GTR+I+G was used and this matrix had 1918 distinct alignment patterns with 49.30% undetermined characters or gaps, and the final ML optimization likelihood value of the best tree was −43,306.257377. The estimated base frequencies were as follows: A = 0.254330, C = 0.209623, G = 0.232160, and T = 0.303887; substitution rates: AC = 1.172145, AG = 2.820942, AT = 1.449869, CG = 0.733730, CT = 4.396602, and GT = 1.000000; gamma distribution shape parameter: α = 0.637676. Based on our multiloci phylogenetic analysis (Figure 1), our isolates were placed as independent lineages with high support values between other species, with one for the isolates URM 9233, URM 9234, and URM 9235 (IQ-TREE-BS = 100%, RAxML-BS = 100%, and BPP = 1) and the other for URM 9236 and URM 9237 (IQ-TREEBS = 100%, RAxML-BS = 100%, and BPP = 1), therefore justifying their statuses as two new species.

#### 3.1.2. *Gongronella*

**Figure 2:**
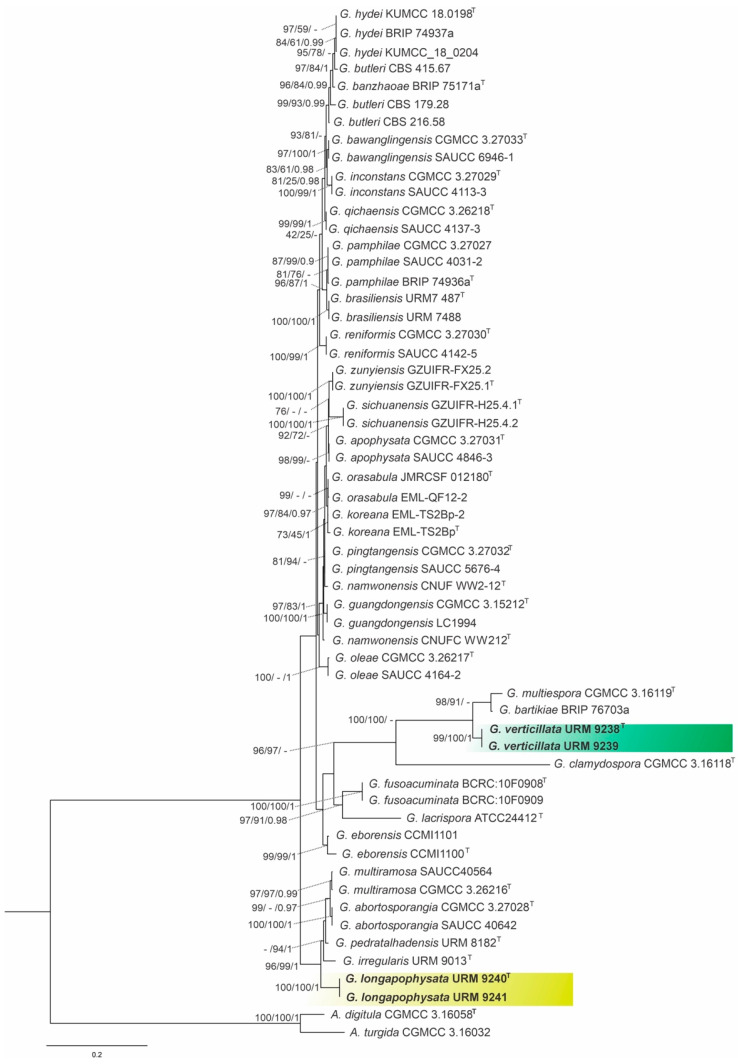
Maximum Likelihood (ML) phylogenetic tree of the combined ITS, LSU, and *act* sequences of *Gongronella* species. The specimens obtained in this study are in bold and highlighted in yellow or green Ex-type strains are marked with (T). Confidence values for ML-BS ≥ 70% (UFboot2/RAxML) and BPP ≥ 0.95 are included near the nodes and the “-” indicates statistical support below the threshold values. The tree was rooted to *Absidia digitula* (CGMCC 3.16058) and *Absidia turgida* (CGMCC 3.16032).

The combined matrix of three markers of *Gongronella* species (ITS, LSU, and *act*) comprised sequences from 58 strains, including the outgroup *Absidia digitula* (CGMCC 3.16058) and *Absidia turgida* (CGMCC 3.16032), with a total of 2426 characters, including gaps, with 760 characters for ITS, 928 for LSU and 738 for *act*. The model used for IQ-TREE ML and BI was GTR+G for both genes. For the ML analysis (RAxML-HPC BlackBox), the model GTR+I+G was used; this matrix had 904 distinct alignment patterns with 35.71% undetermined characters or gaps, and the final ML optimization likelihood value of the best tree was -11879.360036. The estimated base frequencies were as follows: A = 0.250425, C = 0.216708, G = 0.245264, and T = 0.287603; substitution rates: AC = 0.872173, AG = 2.309143, AT = 1.587038, CG = 0.516161, CT = 4.286676, and GT = 1.000000; gamma distribution shape parameter: α = 0.508375. Based on our multiloci phylogenetic analysis (Figure 2), our isolates were placed as independent lineages with high support values between other species, with one for the isolates URM 9240, and URM 9241 (IQ-TREE-BS = 100%, RAxML-BS = 100%, and BPP = 1) and the other for URM 9238 and URM 9239 (IQ-TREEBS = 99%, RAxML-BS = 100%, and BPP = 1), therefore justifying their status as two new species.

### 3.2. Taxonomy

#### 3.2.1. *Absidia rhizoidea* L.W.S. de Freitas & A.L. Santiago, sp. nov. (Figure 3 and Figure 4)

Mycobank number: MB863003.

Etymology: The epitet *rhizoidea* (Lat.), referring to the rhizopodiform rhizoids formed at the end of solons, commonly next to the sporangiophores.

Diagnosis: The rhizoids are strongly branched, rhizopodiform, commonly formed next to the sporangiophores. Chlamydospores abundant.

Description: Colonies floccose on PDA, initially white (A1), turning brown (6E8), 8.5 cm in diameter after 8 days of incubation at 25 °C; reverse caramel brown (6C6), irregular. Rhizoids caramel brown, commonly formed at the end of stolons and very close to the sporangiophores, rhizopodiform, strongly branched, 2.5–4.5 µm in diameter, smooth-walled. Sporangiophores brown, arising from stolons, isolated or in whorls of up to seven, unbranched, simple or rarely sympodially branched up to three times, (7–)20–350(–575) × (2.5–)5–12(–20) µm, smooth-walled, occasionally with one swelling after 7 days of incubation. One septum is observed near the apophysis. Sporangia light brown, subglobose, 12–40 × 10–40 µm, smooth and deliquescent-walled. Apophysis brownish, short or long, cup or bell-shaped, 5–30 × 6–30 µm. Columellae light brown, hemispherical or subglobose, 2.5–17 × 7–22 µm, occasionally with up to two needle-shaped or filiform with bulbous tip projection, 3–12 × 2–4 µm. Sporangiospores hyaline, cylindrical, some with a slight constriction in the center, ellipsoid and subglobose, 3–8 × 2–6 µm, smooth-walled. Chlamydospores abundant, hyaline, globose and subglobose, many attached to rhizoids or formed in aerial hyphae, 2.5–7 µm in diameter. Zygosporangia not observed.

Growth experiments (8 days): On PDA: At 5 °C—no growth; at 10 °C—1.5 cm diameter; at 15 °C—5.5 cm diameter; at 20 °C—8 cm diameter; at 25 °C—8.5 cm diameter; at 30 °C—8.3 cm diameter; at 35 °C—no growth. On MEA: At 5 °C—no growth; at 10 °C—0.7 cm diameter; at 15 °C—5.3 cm diameter; at 20 °C—8.8 cm diameter; at 25 °C—9 cm diameter; at 30 °C—8.5 cm diameter; at 35 °C—no growth. The Tmax is 33 °C on MEA and 32 °C on PDA.

Habitat and Distribution: Soil from Maranhão state (Brazil).

Specimen examined: BRAZIL, Maranhão, Carolina city, Chapada das Mesas, Cachoeira do Dodô (7°05′33.2″ S 47°26′35.5″ W), soil, 15 May 2023, L.W.S. de Freitas (Holotype URM 9234H metabolically inactive state, Ex-type living culture URM 9234).

GenBank accession numbers: ITS = PZ234078, LSU = PZ227107, *act* = PZ227661.

Additional specimens examined: BRAZIL, Maranhão, Carolina, Chapada das Mesas, Cachoeira do Dodô (7°05′33.2″ S 47°26′35.5″ W), soil, 15 May 2023, L.W.S. de Freitas (URM 9233); Encanto azul (7°14′00.6″ S 46°24′24.3″ W), soil, 15 May 2023, L.W.S. de Freitas (URM 9235).

GenBank numbers: URM 9233, ITS = PZ234079, LSU = PZ227108, *act* = PZ227662; URM 9235, ITS = PZ234080, LSU = PZ227109, *act* = PZ227663.

Notes: In our phylogenetic analysis, *Absidia rhizoidea* is a sister species of *A. variiprojecta* (Figure 1). Morphologically, *A. rhizoidea* forms constant rhizoids close to the sporangiophores, in addition to sporangiophores arising in whorls of up to seven from stolon, while *A. variiprojecta* has rhizoids not commonly formed close the sporangiophores and forms sporangiophores arising in whorls of up to five from stolon. Furthermore, columellae of the new species are hemispherical or subglobose, occasionally with up to two needle-shaped or filiform with bulbous tip projection, differing from *A. rhizoidea* that forms columellae subglobose or subglobose to fig-shaped, with one projection generally needle- or triangle-shaped, some bulbous, very rarely feather-shaped. The Tmax of *A. variiprojecta* is 32 °C, both on PDA and MEA [20], while the Tmax values of the new species are 33 and 32° C on MEA and PDA, respectively.

**Figure 3:**
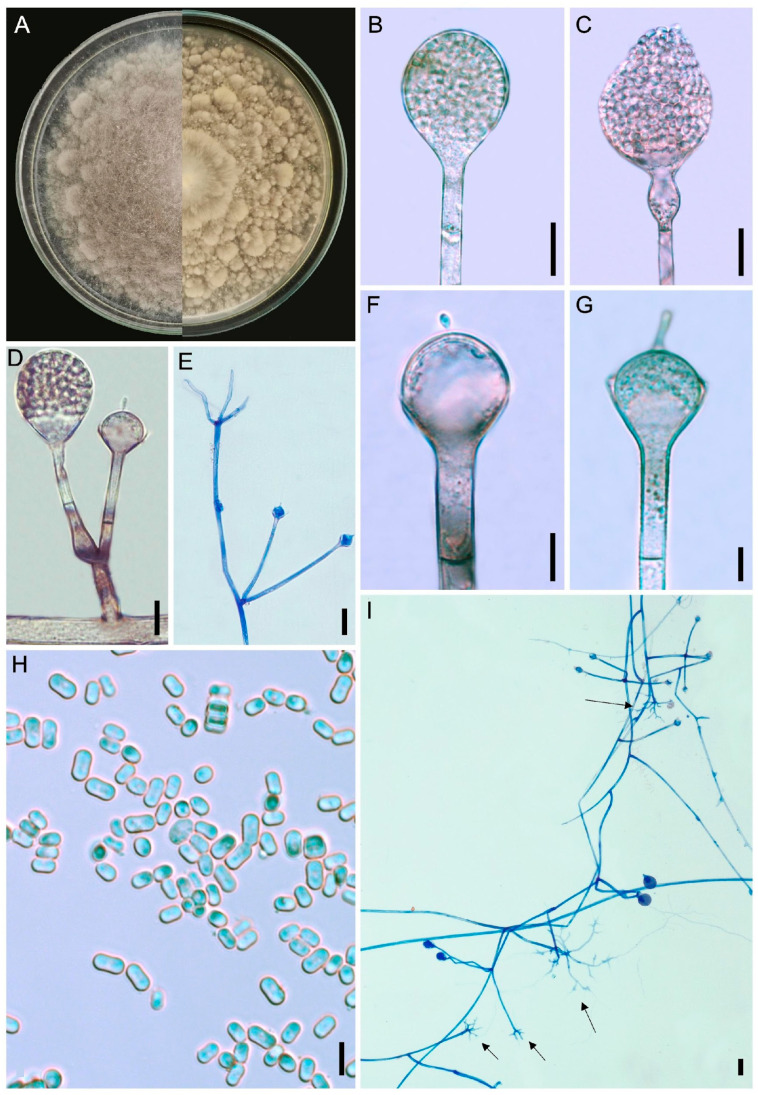
*Absidia rizhoidea* (URM 9234, ex-type) on PDA at 25 °C after eight days. (**A**) Colony verse (left) and reverse (right). (**B**) Sporangiophore with sporangium. (**C**) Sporangiophore with a swallow and sporangium. (**D**) Sporangiophore short branched with sporangium and projection. (**E**) Two sporangiophores arising from stolon with rhizoid. (**F**,**G**) Sporangiophore with apophysis, columella and projection. (**H**) Sporangiospores. (**I**) Stolons with sporangiophores and rhizoids (arrows). Scale bars: (**B**,**C**) 20 μm; (**D**,**E**,**I**) 15 μm; (**F**,**G**) 10 μm; (**H**) 10 μm.

**Figure 4:**
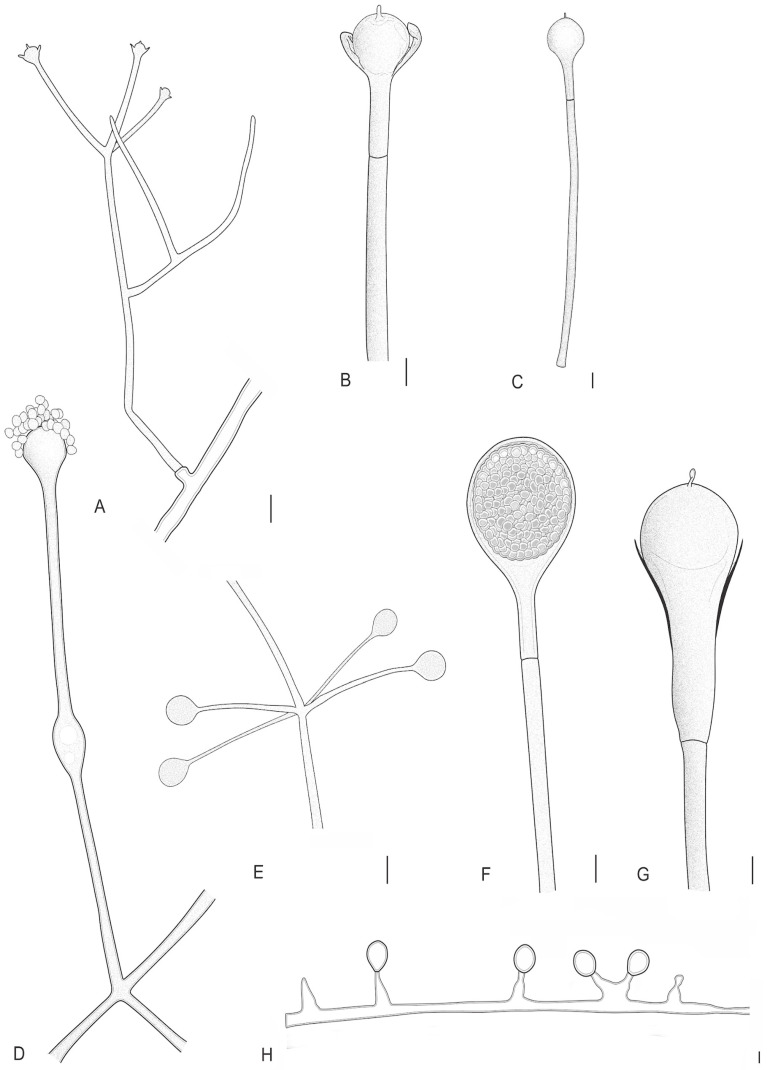
*Absidia variabilis* (URM 9236, ex-type) on PDA at 25 °C after eight days. (**A**) Branched sporangiophore. (**B**,**C**) Sporangiophore with a septum below the apophysis, columella and projection. *Absidia rizhoidea* on PDA at 25 °C after eight days. (**D**) Sporangiophore with swelling. (**E**) Sporangiophores in a whorl of four arising from stolon. (**F**) Sporangiophore with sporangium. (**G**) Sporangiophore with a long apophysis, columella and projection. (**H**) Chlamydospores. Scale bars: (**A**) 5 μm; (**B**,**C**) 8 μm; (**D**,**E**) 20 μm; (**F**–**H**) 10 μm.

#### 3.2.2. *Absidia variabilis* L.W.S. de Freitas & A.L. Santiago, sp. nov. (Figure 4 and Figure 5)

Mycobank number: MB863004.

Etymology: The epitet *variabilis* (lat.) refers to the formation of sporangiophores variable in length.

Diagnosis: Sporangiophores varied in length, arising singular or in whorls of up to five from stolons, commonly unbranched or monopodially branched, never sympodially branched; chlamydospores are abundant.

Description: Colonies floccose on PDA, initially white (A1), becoming light brown (6D8), 8.5 cm in diameter after 8 days of incubation, at 25 °C; reverse light brown (6D8), wave zonate. Rhizoids brown, poorly branched, often with adherent chlamydospores, 2.5–5.5 µm in diameter, with encrusted wall. Sporangiophores erect, light brown, arising from stolons, singular or in whorls of up to five, monopodially, but never sympodially branched, varied in length (15–)60–190(–230) × (2–)3–4(–6) µm, smooth-walled, with no swellings. One septum is observed near the apophysis. Sporangia apophysate, light brown, globose to slightly dorsiventrally flattened, pyriform (when considering the apophysis), (7–)10–20(–30) × (10–)15–30 µm, smooth, with deliquescent wall. Apophysis brownish, short, cup-shaped, (2–)4.5–7(–12) × (3.5–)7.5–15 µm, smooth-walled. Columellae light brown slightly flattened and subglobose (2–)5–17 × (5–)9–22 µm, smooth-walled, collar visible. One filiform, spine-like or occasionally triangular projection, up to 1–4 × 0.5–2.5 µm, can be observed on the columellae surface. Sporangiospores hyaline, mostly cylindrical, constricted in the center, some short cylindrical, 2.5–5.5 × 1.5–2.5 µm, smooth-walled. Chlamydospores abundant, hyaline, globose, subglobose, frequently attached to rhizoids or in aerial hyphae, 2–6 µm in diameter. Zygosporangia not observed.

Growth experiments (8 days): On PDA: At 5 °C—no growth; at 10 °C—1.1 cm diameter; at 15 °C—4.6 cm diameter; at 20 °C—8.6 cm diameter; at 25 °C—9 cm diameter; at 30 °C—8.5 cm diameter; at 35 °C—no growth. On MEA: At 5 °C—no growth; at 10 °C—0.7 cm diameter; at 15 °C—5.1 cm diameter; at 20 °C—8.5 cm diameter; at 25 °C—8.8 cm diameter; at 30 °C—8.6 cm diameter; at 35 °C—no growth. The Tmax is 31 °C on MEA and 32 °C on PDA.

Habitat and Distribution: Soil from Maranhão state (Brazil).

Typification: BRAZIL, Maranhão, Carolina, Chapada das Mesas, São Romão (6°02′09.1′′ S 46°34′03.5′′ W), soil, 15 May 2023, L.W.S de Freitas (Holotype URM 9236H metabolically inactive state, Ex-type living culture URM 9236).

GenBank accession numbers: ITS = PZ234081, LSU = PZ227110, *act* = PZ227664.

Additional specimen examined: BRAZIL, Maranhão, Carolina, Chapada das Mesas, São Romão (6°02′09.1′′ S 46°34′03.5′′ W), soil, 15 May 2023, L.W.S. de Freitas (URM 9237). GenBank accession numbers: ITS = PZ234082, LSU = PZ227111, *act* = PZ227665.

Notes: *Absidia variabilis* is phylogenetically more closely related to *A. variispora* T.R.L. Cordeiro & A.L. Santiago (Figure 1). The main morphological differences between both species are the formation of sporangiophores arising singular or in whorls of up to six from stolon, as well as the formation of cylindrical, elliptical, globose, and subglobose sporangiospores by *A. variispora*, while *A. variabilis* forms sporangiophores single or in whorls of up to five from stolon and produces exclusively cylindrical spores. Furthermore, *A. variabilis* forms chlamydospores, while *A. variispora* does not. *Absidia variabilis* grows up to 31 °C on MEA and 32 °C on PDA, while *A. variispora* can grow at 32 °C on MEA and PDA [20].

**Figure 5:**
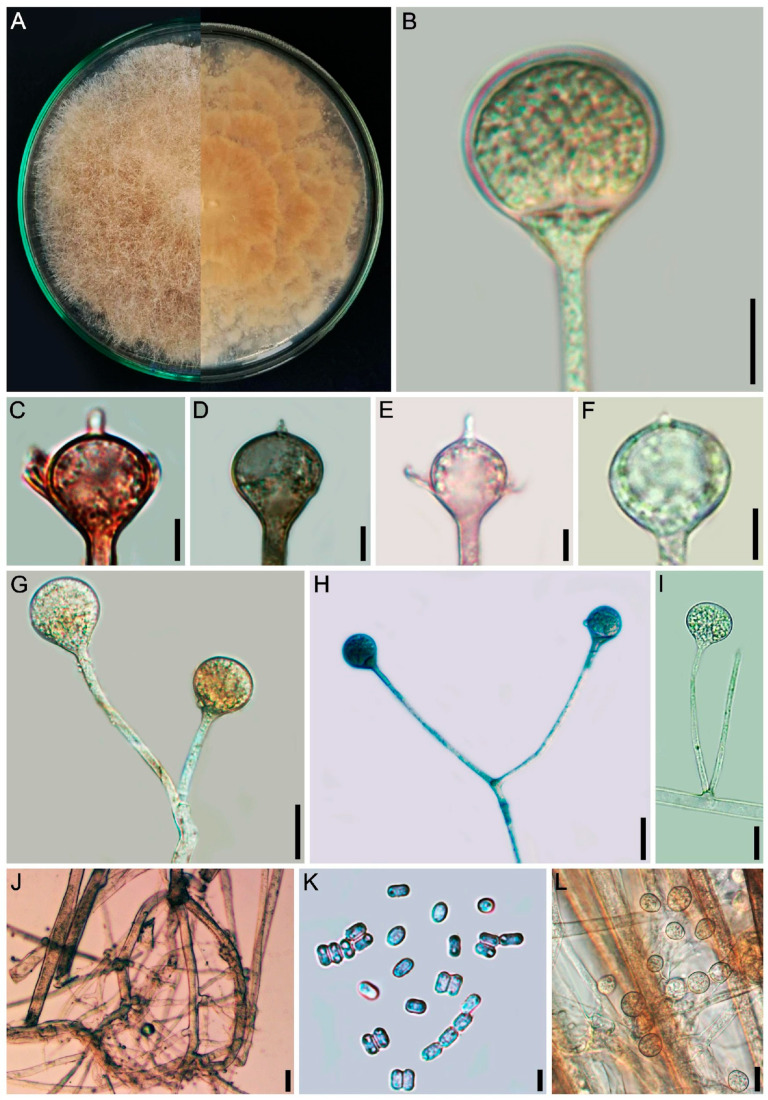
*Absidia variabilis* (URM 9236, ex-type) on PDA at 25 °C after eight days. (**A**) Colony verse (left) and reverse (right). (**B**) Sporangiophore with sporangium. (**C**–**F**) Different types of columellae, apophyses, and projections. (**G**,**H**) Branched sporangiophore. (**I**) Two sporangiophores arising from stolon. (**J**) Rhizoid. (**K**) Sporangiospores. (**L**) Chlamydospores. Scale bars: (**B**) 15 μm; (**C**–**I**) 10 μm; (**J**–**L**) 5 μm.

#### 3.2.3. *Gongronella longapophysata* L.W.S de Freitas & A.L. Santiago, sp. nov. (Figure 6 and Figure 7)

Mycobank number: MB863005.

Etymology: The epithet *longapophysata* (Lat.) refers to the sporangiophores with elongated apophysis.

Diagnosis: The species forms sporangiophores with some very elongated apophyses, some with an elongated swelling below apophyses, and chains of 3 to 12 sterile sporangia along sporangiophores.

Description: Colonies white (A1), cottony, with regular margin, 6.5 cm in diameter on PDA after 8 days, at 25 °C. Reverse white (A1). Rhizoids branched, long, often with adherent chlamydospores, 2–10 μm in diameter, and bulbous, 5–38 μm in diameter. Stolons hyaline, branched and coenocytic. Sporangiophores hyaline, erect, curved to sinuous, (10–)35–230(–350) × 2.5–7(–12) μm, with one to two septa below the sporangium, simple or sympodially branched up to four times, wall smooth to slightly incrusted, rarely verticillately branched in whorls of up to five. Fertile sporangia hyaline, subglobose (5–)12–20(–25) × (6.5–)15–20(–25) μm, wall with vitreous aspect, smooth and deliquescent, leaving a collar. Sterile sporangia hyaline, ellipsoid, cylindrical to constricted frequently formed on short sporangiophores branches, 3–20 μm in diameter, occasionally so short that the sterile sporangia appear to be sessile; 3–6, rarely, 7–12 sterile sporangia are also formed along the sporangiophore. Columellae hyaline, hemispherical, subglobose (2–)5–10 × (3.5–)7–11(–15) μm. Apophyses bell-shaped, long or short, vasiform, (2–)3–7(–10) × (5–)7–10(–13) μm. Some sporangiophores may show an elongated swelling below the apophysis, 10–25 × 5–7 μm. Sporangiospores hyaline, reniform, some irregularly-shaped, 2–5.5 × 1.5–2 μm, smooth-walled. Chlamydospores abundant, hyaline, globose, 4–15 μm in diameter. Zygosporangia not observed.

Growth experiments (8 days): On PDA: At 10 °C—no growth; at 15 °C—1.8 cm diameter; at 20 °C—3.1 cm diameter; at 25 °C—6.3 cm diameter; at 30 °C—7.5 cm diameter; at 35 °C—no growth. On MEA: At 10 °C—no growth; at 15 °C—1.9 cm diameter; at 20 °C—4.4 cm diameter; at 25 °C—6.0 cm diameter; at 30 °C—6.8 cm diameter; at 35 °C—no growth. The Tmax is 32 °C on MEA and 33 °C on PDA.

Habitat and Distribution: Soil from Maranhão state (Brazil).

Specimen examined: Brazil, Maranhão, Carolina city, Chapada das Mesas, Cachoeira da Mansinha (7°07′54.0″ S 47°26′55.1″ W), soil, 15 May 2023, L.W.S. de Freitas (Holotype URM 9240H metabolically inactive state, Ex-type living culture URM 9240).

GenBank accession numbers: ITS = PZ234083, LSU = PZ227112, *act* = PZ227666.

Additional specimen examined: BRAZIL, Maranhão, Carolina city, Chapada das Mesas, Cachoeira da Mansinha (7°07′54.0″ S 47°26′55.1″ W), soil, 15 May 2023, L.W.S de Freitas (URM 9241).

GenBank accession numbers: ITS = PZ234084, LSU = PZ227113, *act* = PZ227667.

Notes: *Gongronella longapophysata* is phylogenetically closely related to *G. multiramosa* and *G. abortosporangia* (Figure 1). Morphologically, *G. multiramosa* and *G. abortosporangia* can be easily differentiated from *G. longapophysata* due to no formed stolons, no verticillately branched sporangiophores (rare in *G. longapophysata*), no sporangiophores with a swelling below apophysis, and no chains of 3 to 12 sterile sporangia along sporangiophores [30]. The Tmax of *G. longapophysata* is 32 °C on MEA and 33 °C on PDA, but Tmax of *G. abortosporangia* and *G. multiramosa* is not indicated in the protologues nor elsewhere [29,30].

**Figure 6:**
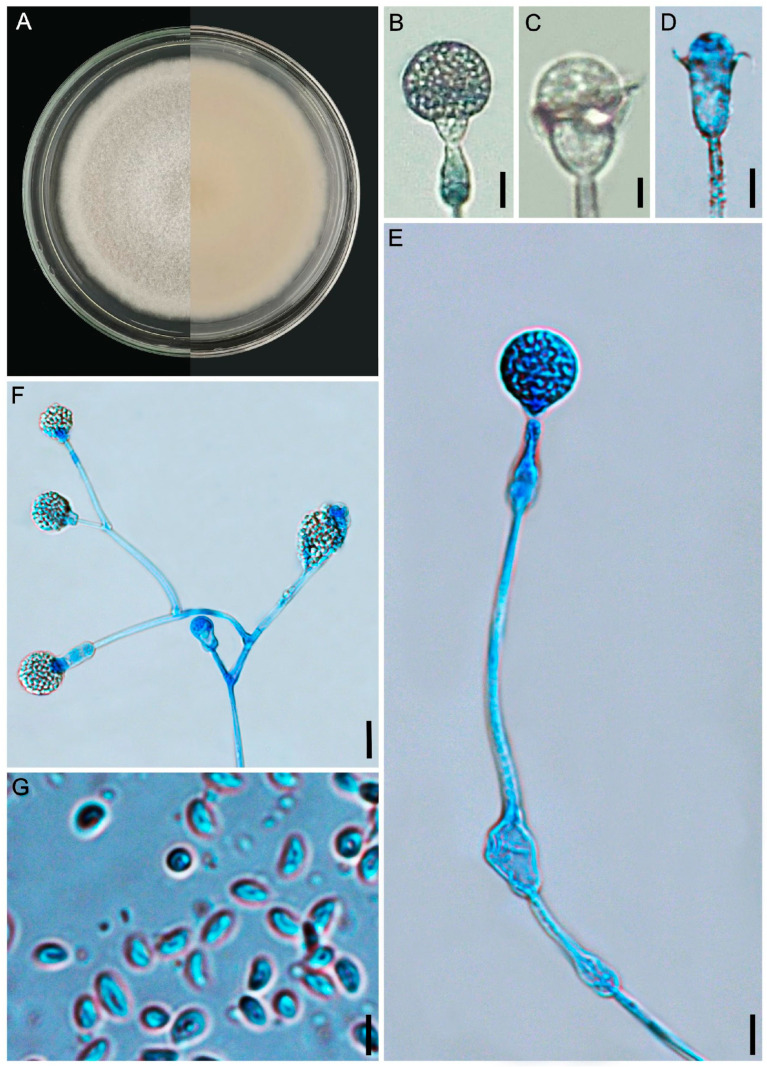
*Gongronella longapophysata* (URM 9240, ex-type) on PDA at 25 °C after eight days. (**A**) Colony verse (left) and reverse (right). (**B**–**D**) Columellae and aphophyses. (**E**) Sporangiophore with elongated swelling below the apophysis and sterile sporangia. **(F**) Branched sporangiophore. (**G**) Sporangiospores. Scale bars: (**B**–**E**) 10 μm; (**F**,**G**) 5 μm.

**Figure 7:**
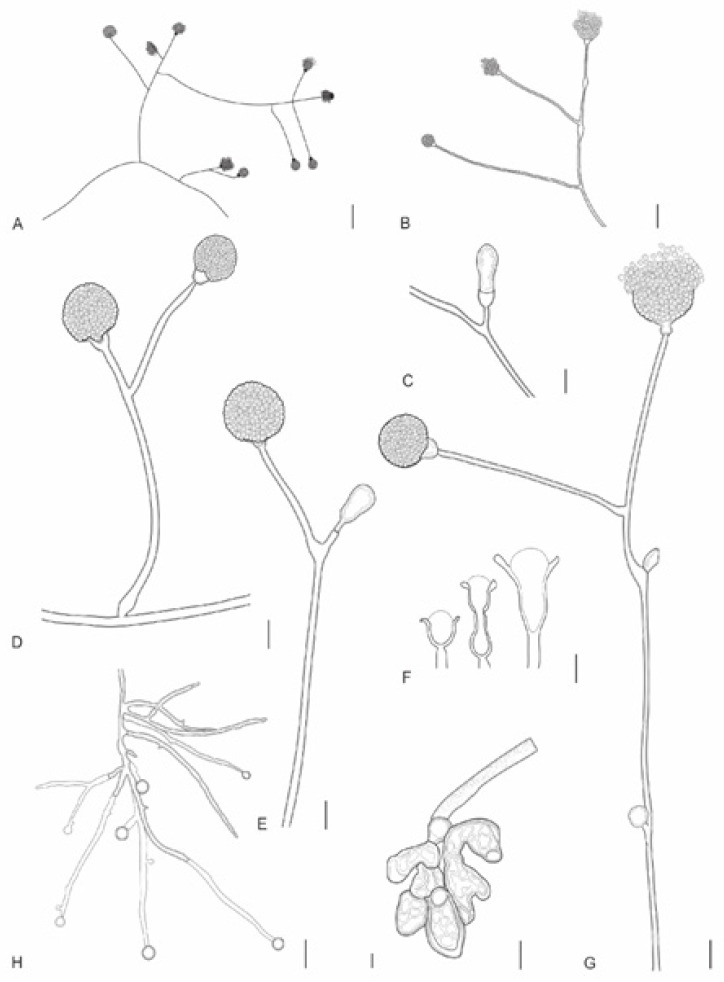
*Gongronella longapophysata* (URM 9240, ex-type) on PDA at 25 °C after eight days. (**A**,**B**) Branched sporangiophore with fertile sporangia. (**C**) Sterile sporangium. (**D**) Simply branched sporangiophore with sporangia. (**E**) Simply branched sporangiophore with fertile (terminal) and sterile (branch) sporangia. (**F**) Apophyses and columellae. (**G**) Sporangiophore with long and short branches. (**H**) Rhizoid with chlamydospores. (**I**) Bulbous rhizoid. Scale bars: (**A**,**B**) 30 μm; (**C**–**F**) 5 μm; (**G**–**I**) 10 μm.

#### 3.2.4. *Gongronella verticillata* L.W.S. de Freitas & A.L. Santiago, sp. nov. (Figure 8 and Figure 9)

Mycobank number: MB863006.

Etymology: The epithet *verticillate* (Lat.) refers to the formation of sporangiophores verticillately branched.

Diagnosis: The species commonly forms sporangiophores branched in whorls with up to five branches.

Description: Colonies white (A1), cottony, regular margin, 7 cm in diameter on PDA after 8 days of incubation, at 25 °C; reverse cream (4A3). Rhizoids of two types: root-shaped, hyaline, branched, with spaced septa and chlamydospores attached, and bulbous, without chlamydospores attached, 2.5–8.5 µm in diameter. Stolons hyaline, simple and coenocytic. Sporangiophores simple or sympodially branched up to six times, and commonly verticillately branched, with up to five branches in a whorl, (25–)95–200(–250) × 2.5–5 μm, with one or two septa below the sporangium, rarely forming three septa, smooth-walled. Some sporangiophores are extremely short, 2.5–10 × 2–3 μm, forming sterile apical or lateral sporangia. Fertile sporangia hyaline, globose, (6–)10–17(–22) μm diameter, with vitreous aspect, smooth and deliquescent-walled, leaving a collar. Abortive sporangia ovoid, subglobose and globose 4–18 μm in diameter. Columellae hyaline, mostly globose 1–5 μm in diameter, subglobose, hemispherical, flattened and rounded-topped (1–)4–7(–9) × (2–)6–9(–12) μm, smooth-walled, occasionally so flattened that they seem inconspicuous. Apophyses hyaline, mostly globose, 3–10 μm diameter, subglobose, some bell-shaped and vasiform, (3–)5–10(–13) × (3.5–)6–12(–15) μm, smooth-walled. Sporangiospores hyaline, elliptical to fusiform, irregular or reniform, 2–7.5 × 1.5–4 μm, smooth-walled. Chlamydospores abundant, hyaline, subglobose and ellipsoidal, 4–15 μm diameter. Zygosporangia not observed.

Growth experiments (8 days): On PDA: At 10 °C—no growth; at 15 °C—1.7 cm diameter; at 20 °C—3.2 cm diameter; at 25 °C—6.8 cm diameter; at 30 °C—2.1 cm diameter; at 35 °C—no growth. On MEA: At 10 °C—no growth; at 15 °C—1.9 cm diameter; at 20 °C—4.2 cm diameter; at 25 °C—5.5 cm diameter; at 30 °C—4 cm diameter; at 35 °C—no growth. The Tmax is 31 °C on both MEA and PDA.

Habitat and Distribution: Soil from Maranhão state (Brazil).

Specimen examined: BRAZIL, Maranhão, Carolina city, Chapada das Mesas, Cachoeira da Mansinha (7°07′54.0″ S 47°26′55.1″ W), soil, 15 May 2023, L.W.S. de Freitas (Holotype URM 9238H metabolically inactive state, Ex-type living culture URM 9238).

GenBank accession numbers: ITS = PZ234085, LSU = PZ227114.

Additional specimen examined: BRAZIL, Maranhão, Carolina city, Chapada das Mesas, Cachoeira da Mansinha (7°07′54.0″ S 47°26′55.1″ W), soil, 15 May 2023, L.W.S. de Freitas (URM 9239).

GenBank accession numbers: ITS = PZ234086, LSU = PZ227115.

Notes: *Gongronella verticillata* is phylogenetically closely related to *G. multispora* and *G. bartikiae* (Figure 1). However, *G. verticillata* forms sporangiophores sympodially and verticillately branched, with up to five branches in a whorl. The apophyses are globose, subglobose, bell-shaped and vasiform; columellae are globose, subglobose, flattened, rounded-topped and inconspicuous, whereas *G. multispora* forms sporangiophores sympodially and verticillately branched, with 2–3 branches in a whorl. Apophyses are pyriform to subglobose, and the columellae are hemispheric and some inconspicuous. It is curious that both species form sporangiophores branched in whorls, as to the best of our knowledge, this branch pattern has also been reported in *G. namwonensis* so far [28]. The Tmax of *G. verticillata* is 31 °C on MEA and PDA, but Tmax of *G. multispora* has not been given in the protologue [29]. There is no morphological description of *G. bartikiae* in the literature for comparison [67].

**Figure 8:**
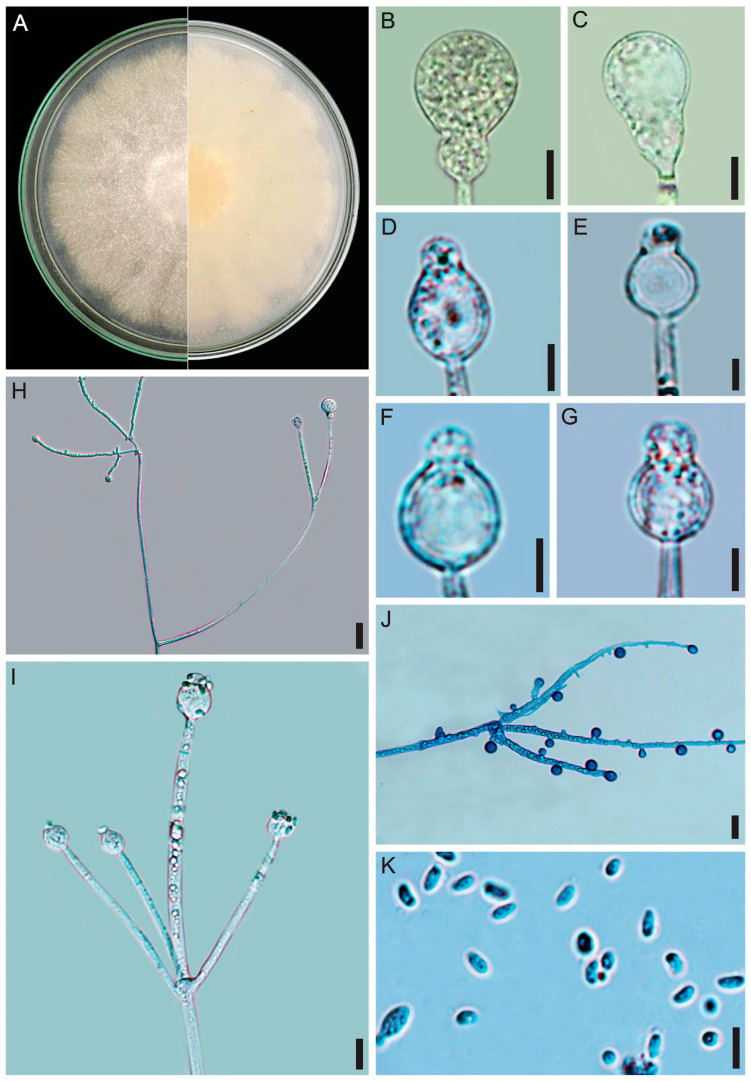
*Gongronella verticillata* (URM 9238, ex-type) on PDA at 25 °C after eight days. (**A**) Colony verse (left) and reverse (right). (**B**,**C**) Sporangiophore with sporangium. (**D**–**G**) Sporangiophore with columella. (**H**) Simply branched sporangiophore arising from stolon with rhizoid. (**I**) Sporangiophore with terminal columella and lateral whorled branches with columellae. (**J**) Rhizoid with chlamydospores attached. (**K**) Sporangiospores. Scale bars: (**B**,**C**) 20 μm; (**D**,**F**,**J**,**K**) 10 μm; (**G**,**H**) 15 μm; (**E**,**I**) 5 μm.

**Figure 9:**
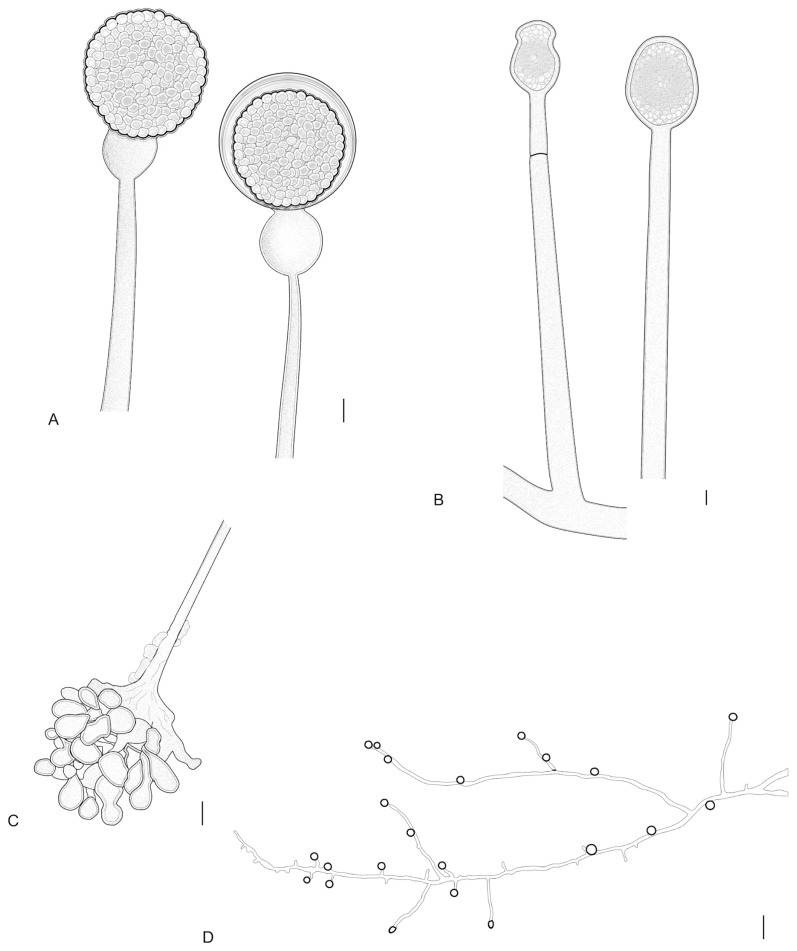
*Gongronella verticillata* (URM 9238, ex-type) on PDA at 25 °C after eight days. (**A**) Sporangiophores with sporangia. (**B**) Sporangiophores with a septum below the apophysis (left) and columellae. (**C**) =Bulbous rhizoid. (**D**) Chlamydospores attached to the mycelium. Scale bars: (**A**,**B**) 3 μm; (**C**) 10 μm; (**D**) 5 μm.

## 4. Discussion

Traditionally, new species of *Mucorales* fungi have been mostly delimited on a morphological basis, leading to further synonymies and generating controversy and doubts among taxonomists. In this context, species of *Absidia* and *Gongronella* have been sorted into other genera, as they shared similar morphological characteristics. For instance, *G. butleri* was first described as *A. butleri* Lend. in 1926, while Paine [68] proposed *A. subpoculata* Paine, which was later synonymized with *G. butleri*. In addition, some species of *Absidia* have been previously included in *Tieghemella*, *Mycocladus*, *Proabsidia*, *Pseudoabsidia*, and *Protoabsidia* [69,70,71]. On the other hand, species of *Lichtheimia* and *Lentamyces* have been previously treated as *Absidia* [72,73].

The advent of the molecular biology allowed the delimitation of new species of *Absidia* and *Gongronella* in the last 10–15 years in different countries, such as China, Brazil, South Korea, and Thailand, most of which were proposed based in sequences of ITS and/or LSU rDNA [20,21,29,30,41,42,74,75]. Wang et al. [30] highlighted that the ITS, LSU, Elongation Factor 1-alpha-EF-1α (*TEF*), *act*, and RNA Polymerase II Largest Subunit (*RPB1*) are reliable markers for identification of *Gongronella* species. Until 1969, only two *Gongronella* species, namely, *G. butleri* and *G. lacrispora* Hesselt. & J. JEllis [76,77], were known, and this number has increased to 28 species (as of 3 March 2026), mostly descried from China. Regarding *Absidia*, Ji et al. [44] sequenced *TEF* and *act*, as well as ITS and LSU, to delimit new species, and 69 new species have been described in the last 15 years [27].

Since Tmax has been successfully used as a discriminative characteristic for delimitation of mucoralean fungi, such as *Backusella* [39], *Cunninghamella* [78,79], *Lichtheimia* [80], *Mucor* [81], and *Rhizopus* [82], we believe in the taxonomic relevance of this characteristic for the delimitation of species within *Absidia* and *Gongronella*. In this paper, the Tmax of our new *Absidia* species did not vary much from the phylogenetically close species. However, we retrieved data from the literature on the Tmax of 50 species of *Absidia* (plus two new species described in this work) and realized that the Tmax varies considerably between species in this genus, from 24 °C (*A. frigida*) to 37 °C (*A. ovalispora* and *A. zonata*) (Table 1); therefore, this feature has taxonomic value. However, as it was also observed in other mucoralean genera [39,78,79,80], some overlapping may occur (e.g., *A. menglianensis*, *A. tarda* and *A. longissima* have a Tmax of 36 °C), which is why this feature should be used along with morphology and phylogeny for delimiting species in *Absidia*, as well as in other mucoralean genera.

We were unable to compare the Tmax of our new *Gongronella* species with other species due to the lack of Tmax data in the literature for this genus; therefore, it is still unknown whether this feature is taxonomically informative in *Gongronella*. Nevertheless, to the best of our knowledge we are providing the first data on Tmax for the genus. Regarding *Absidia*, Tmax data are available in the literature for only 52% of the known species, mostly for species from China and Korea, with a few species from Brazil and Australia (Table 1). Therefore, studies evaluating the Tmax of all species of both genera should be encouraged.

In this paper, we described, based on an integrated approach, four new species of *Cunninghamellaceae* from soil samples from the still poorly studied Cerrado biome. As far as we are aware, this is the first report of new *Absidia* and *Gongronella* species in this biome. Currently, only 17 species of *Absidia* have been reported in Brazil, including ten new species [19,20,53,74,75,83,84], whereas only four species of *Gongronella* were recorded in the country, including three new species [85,86]. All these species were isolated in the Caatinga and Atlantic Forest biomes. This paper increases the number of known species of *Absidia* and *Gongronella* to 96 and 30, respectively, as well as the knowledge of these fungi in the Brazilian Savanna. We believe that new inventories of *Cunninghamellaceae* in other Cerrado areas may unveil additional new species.

## Figures and Tables

**Table 2:** Molecular markers used in this study with respective primer pairs and PCR protocols.

Markers	PCR Primers	PCR Conditions *	References
ITS	ITS4/ITS5	95 °C, 5 min; (95 °C, 30 s; **55 °C**, 45 s; 72 °C, 1:15 min) × 35 cycles; 72 °C, 10 min	White et al. [54]
LSU	LR5/LR0R	95 °C, 5 min; (95 °C, 30 s; **55 °C**, 45 s; 72 °C, 1:15 min) × 35 cycles; 72 °C, 10 min	Vilgalys and Hester [55]; Rehner and Samuels [56]
*act*	Act-1/Act-4R	95 °C, 3 min; (95 °C, 60 s; **55 °C**, 60 s; 72 °C, 60 s) × 30 cycles; 72 °C, 10 min	Voigt and Wöstemeyer [18]

* Annealing temperatures are in bold.

**Table 3:** GenBank accession numbers of sequences used in this study. Sequences in boldface were obtained during this study. Ex-type strains are marked with (T).

Species	Strains	GenBank Accession Numbers		
		ITS	LSU	*act*
*Absidia abundans*	XY09265	ON074697	ON074681	–
*Absidia abundans*	CGMCC 3.16255^T^	NR_182590	ON074683	–
*Absidia abundans*	XY09274	ON074696	ON074682	–
*Absidia aguabelensis*	URM 8213^T^	NR_189383	NG_241934	–
*Absidia alpina*	CGMCC 3.16104	OL678133	–	–
*Absidia ampullacea*	CGMCC 3.16054	MZ354138	MZ350132	–
*Absidia anomala*	CBS 125.68^T^	MH859085	MH870799	–
*Absidia anomala*	FSU5798	EF030523	–	EF030535
*Absidia arrhiza*	CGMCC 3.28540^T^ = XG09770-7	PQ600860	PQ600254	PQ753533
*Absidia arrhiza*	XG09770-6	PQ600859	PQ600253	PQ753532
*Absidia biappendiculata*	CBS 187.64	MZ354153	MZ350147	MZ357438
*Absidia bonitoensis*	URM 7889^T^	MN977786	MN977805	–
*Absidia brunnea*	CGMCC 3.16055^T^	MZ354139	MZ350133	MZ357421
*Absidia brunneola*	CGMCC 3.29490^T^ = XG12984-12-1	NMDCN0009VE 6	NMDCN000 9VEM	NMDCN0009 TVO
	XG12984-12-2	NMDCN0009VE 7	NMDCN000 9VEN	NMDCN0009 TVP
*Absidia caatinguensis*	URM 7156^T^	NR_154704	NG_058582	–
*Absidia caerulea*	XY00608	OL620081	–	–
*Absidia caerulea*	XY00729	OL620082	–	–
*Absidia caerulea*	CBS101.36	MH855718	MH867230	–
*Absidia caerulea*	FSU767	AY944870	–	–
*Absidia californica*	CBS 314.78	JN205816	MH872902	–
*Absidia californica*	FSU4748	AY944873	EU736301	EU736224
*Absidia californica*	CBS 126.68 = FSU4747^T^	AY944872	EU736300	AY944758
*Absidia cheongyangensis*	CNUFC CY2203^T^	PP844904	PP852788	PP893196
*Absidia cheongyangensis*	CNUFC CY2401	–	PP852702	PP893197
*Absidia chinensis*	CGMCC 3.16057	MZ354141	MZ350135	MZ357422
*Absidia chinensis*	CGMCC 3.16056^T^	MZ354140	MZ350134	–
*Absidia cinerea*	CGMCC 3.16062	MZ354146	MZ350140	MZ357427
*Absidia collariata*	CGMCC 3.28536^T^ = XG08666-10-1	PQ610533	PQ605104	PQ613279
*Absidia collariata*	XG08666-10-2	PQ610534	PQ605105	PQ613280
*Absidia cornuta*	URM 6100^T^	NR_172976	MN625255	–
*Absidia crystalloides*	CGMCC3.27496^T^ = SAUCC6948-15	PP377803	PP373736	PP790582
*Absidia crystalloides*	SAUCC693201	PP377804	PP373736	PP790581
*Absidia cuneospora*	CBS 102.59	JN205819	–	–
*Absidia cylindrospora*	CBS 100.08	JN205822	JN206588	–
*Absidia digitata*	CGMCC 3.29492^T^ = XG18784-2-1	NMDCN0009VE 2	NMDCN000 9VEI	NMDCN0009 TVK
	XG18784-2-2	NMDCN0009VE 3	NMDCN000 9VEJ	NMDCN0009 TVL
*Absidia digitula*	CGMCC 3.16058^T^	MZ354142	MZ350136	MZ357423
*Absidia edaphica*	MFLUCC 20-0088	NR_172305	NG_075367	MT410739
*Absidia exilis*	CGMCC 3.29493^T^ = XG21013-11-1	NMDCN0009VE 0	NMDCN000 9VEG	NMDCN0009 TVI
	XG21013-11-2	NMDCN0009VE 1	NMDCN000 9VEH	NMDCN0009 TVJ
*Absidia fluvi*	CNUFC CY2240^T^	PP844891	PP852703	PP893201
*Absidia fluvi*	CNUFC CY2241	PP844892	PP852704	PP893202
*Absidia fluvi*	CNUFC CY2315	PP844893	PP852705	PP893203
*Absidia frigida*	CGMCC 3.16201^T^	NR_182565	OM030223	–
*Absidia fusca*	CBS 102.35^T^	NR_103625	NG_058552	–
*Absidia gemella*	CGMCC 3.16202^T^	OM108488	OM030224	–
*Absidia glauca*	CBS 129233	MH865253	MH876693	–
*Absidia glauca*	CBS 101.08^T^	MH854573	MH866105	–
*Absidia glauca*	FSU660	AY944879	EU736302	EU736225
*Absidia globospora*	CGMCC 3.16031^T^	NR_189829	MW671544	MZ357431
*Absidia globospora*	CGMCC 3.16035	MW671538	MW671545	MZ357432
*Absidia globospora*	CGMCC 3.16036	MW671539	MW671546	MZ357433
*Absidia healeyae*	UoMAU1	MT436028	MT436027	MW861731
*Absidia heterospora*	SHTH021	JN942683	JN982936	NA
*Absidia hainanensis*	CGMCC 3.28535^T^ = XG06908-1	PQ610537	PQ605108	PQ613283
*Absidia hainanensis*	XG06908-4	PQ610538	PQ605109	PQ613284
*Absidia jiangxiensis*	CGMCC 3.16105^T^ = Ab-216	OL678134	PP780377	PP790577
*Absidia jindoensis*	CNUFC-PTI1-1	MF926622	MF926616	MF926510
*Absidia koreana*	EML-IFS45-1^T^	KR030062	KR030056	KR030058
*Absidia koreana*	XY00816	OL620083	ON123771	–
*Absidia koreana*	XY00596	OL620084	–	–
*Absidia kunryangriensis*	CNUFC CY2230	PP844905	PP956882	PP893198
*Absidia lobata*	CGMCC 3.16256	ON074690	ON074679	–
*Absidia longissima*	CGMCC 3.16203^T^	NR_182566	OM030225	–
*Absidia macrospora*	FSU4746	AY944882	EU736303	AY944760
*Absidia medulla*	CGMCC 3.16034	NR_189832	MW671549	MZ357436
*Absidia menglianensis*	KUNCC 24-18541 = root2-17	PQ594927	PQ594929	–
*Absidia menglianensis*	KUNCC 24-18542 = MLAS011	PQ594928	PQ594930	–
*Absidia montepascoalis*	URM 8218^T^	NR_172995	–	–
*Absidia multispora*	URM 8210^T^	MN953780	MN953782	–
*Absidia nigra*	CBS 127.68^T^	NR_173068	MZ350146	MZ357437
*Absidia nigra*	CGMCC 3.16059	MZ354143	MZ350137	MZ357424
*Absidia nigra*	CGMCC 3.16060	MZ354144	MZ350138	MZ357425
*Absidia oblongispora*	CGMCC 3.16061^T^	MZ354145	MZ350139	MZ357426
*Absidia ovalispora*	CGMCC 3.16019^T^	NR_176748	MW264131	–
*Absidia pacifica*	SAUCC6955-16^T^ = CGMCC 3.27497	PP377802	PP373735	PP790579
*Absidia pacifica*	SAUCC6955-15 = SAUCC 413601	PP377801	PP373734	PP790580
*Absidia panacisoli*	SYPF 7183^T^	MF522181	MF522180	–
*Absidia paracylindrospora*	CNUFC L2207	PP844907	PP956883	PP893204
*Absidia pararepens*	XY00631	OL620085	ON123774	–
*Absidia pararepens*	XY00615	OL620086	–	–
*Absidia pararepens*	XY05899	OL620087	–	–
*Absidia pararepens*	CBS 146,002 = CCF 6352^T^	MT193669	MT192308	–
*Absidia pateriformis*	CGMCC 3.27495 = SAUCC6347D-1^T^	PP377805	PP373738	PP790583
*Absidia pateriformis*	SAUCC6347D-2 = SAUCC 634702	PP377806	PP373739	PP790584
*Absidia pernambucoensis*	URM 7219^T^	MN635568	MN635569	–
*Absidia pseudocylindrospora*	EML-FSDY6-2	KU923817	KU923814	KU923815
*Absidia psychrophilia*	FSU 4745	AY944874	EU736306	AY944762
*Absidia purpurea*	CGMCC 3.16106	OL678135	–	–
*Absidia pyriformis*	CGMCC 3.28538^T^ = XG09540-14-1	PQ610531	PQ605102	PQ613277
*Absidia pyriformis*	XG09540-14-5	PQ610532	PQ605103	PQ613278
*Absidia radiata*	CGMCC 3.16257	ON074698	ON074684	–
*Absidia radiata*	XY09330-1	ON074699	ON074685	–
*Absidia repens*	CBS 115583^T^	NR_103624	NG_058551	–
***Absidia rhizoidea***	**URM 9234^T^**	**PZ234078**	**PZ227107**	**PZ227661**
***Absidia rhizoidea***	**URM 9233**	**PZ234079**	**PZ227108**	**PZ227662**
***Absidia rhizoidea***	**URM 9235**	**PZ234080**	**PZ227109**	**PZ227663**
*Absidia saloaensis*	URM 8209^T^	MN953781	MN953783	–
*Absidia sichuanensis*	CGMCC 3.16258^T^	NR_182589	ON074688	–
*Absidia simplex*	CGMCC 3.28541^T^ = XG10012-9	PQ600862	PQ600256	PQ686230
*Absidia simplex*	XG10012-8	PQ600861	PQ600255	PQ686229
*Absidia soli*	MFLU-20-0414^T^	MT396373	MT393988	–
*Absidia sphaerica*	CGMCC 3.28542^T^ = XY00690	PQ600866	PQ600260	PQ777149
*Absidia sphaerica*	XY00690-1	PQ600865	PQ600259	PQ777148
*Absidia spinosa*	FSU551	AY944887	EU736307	EU736227
*Absidia stercoraria*	EML-DG8-1^T^	KU168828	KT921998	KT922000
*Absidia sympodialis*	CGMCC 3.16063^T^	MZ354147	MZ350141	–
*Absidia sympodialis*	CGMCC 3.16064	MZ354148	MZ350142	–
*Absidia tarda*	URM 8412	PP844911	PP956884	PP893199
*Absidia tardiva*	CGMCC 3.28537^T^ = XG08757-4	PQ610529	PQ605100	PQ613275
*Absidia tardiva*	XG08757-6	PQ610530	PQ605101	PQ613276
*Absidia terrestris*	ERP-2017^T^	LT795003	LT795005	–
*Absidia tibetensis*	CGMCC 3.28534^T^ = XG00415-1	PQ610535	PQ605106	PQ613281
*Absidia tibetensis*	XG00415-3	PQ610536	PQ605107	PQ613282
*Absidia thailandica*	MFLUCC:20-0089 = MFLUCC:23-0073^T^	OR606547	OR606546	NA
*Absidia tumida*	CGMCC 3.29491^T^ = XG18709-9-1	NMDCN0009VE 4	NMDCN000 9VEK	NMDCN0009 TVM
*Absidia tumida*	XG18709-9-2	NMDCN0009VE 5	NMDCN000 9VEL	NMDCN0009 TVN
*Absidia turgida*	CGMCC 3.16032^T^	NR_189830	NG_241931	MZ357434
*Absidia varians*	CGMCC 3.16065^T^	MZ354149	MZ350143	MZ357428
*Absidia edaphica*	MFLUCC 20-0088^T^	NR_172305	NG_075367	MT410739
***Absidia variabilis***	**URM 9236^T^**	**PZ234081**	**PZ227110**	**PZ227664**
***Absidia variabilis***	**URM 9237**	**PZ234082**	**PZ227111**	**PZ227665**
*Absidia variiprojecta*	URM 8620^T^	PP844913	PP956885	PP893200
*Absidia variispora*	URM 8720	PP844915	PP956886	PP893205
*Absidia variicolumellata*	URM 8216^T^ = DXL-2021b	MZ331545	MZ331547	–
*Absidia virescens*	CGMCC 3.16067	MZ354151	MZ350145	MZ357430
*Absidia viridis*	CGMCC 3.28539^T^ = XG09563-3	PQ600864	PQ600258	PQ753531
*Absidia viridis*	XG09563-2	PQ600863	PQ600257	PQ753530
*Absidia xinjiangensis*	CGMCC 3.16107^T^	OL678136	–	–
*Absidia yunnanensis*	XY09528	ON074701	ON074686	–
*Absidia yunnanensis*	CGMCC 3.16259^T^	NR_182591	NG_149054	–
*Absidia zonata*	CGMCC 3.16033^T^	NR_189831	MW671548	MZ357435
*Absidia zygospora*	RSPG 214	KC478527	–	–
*Absidia zygospora*	ANG28	DQ914420	–	–
*Gongronella abortosporangia*	CGMCC 3.27028^T^	PP195847	PP195948	PP933938
*Gongronella abortosporangia*	CGMCC 3.27028^T^	PP195848	PP195949	PP933939
*Gongronella apophysata*	SAUCC 4846-3	PP195854	PP195955	PP933948
*Gongronella apophysata*	CGMCC 3.27031^T^	PP195853	PP195954	PP933947
*Gongronella banzhaoae*	BRIP 75171a^T^	OR271908	OR259049	–
*Gongronella bartikiae*	BRIP 76703a	PQ882522	–	–
*Gongronella bawanglingensis*	CGMCC 3.27033^T^	PP195857	PP195958	PP933951
*Gongronella bawanglingensis*	SAUCC 6946-1	PP195858	PP195959	PP933952
*Gongronella brasiliensis*	URM 7487^T^	NR_155148	KY114932	–
*Gongronella brasiliensis*	URM 7488	KY114931	KY114933	–
*Gongronella butleri*	CBS 216.58	JN206285	MH869292	–
*Gongronella butleri*	CBS 179.28	JN206286	–	–
*Gongronella butleri*	CBS 415.67	MH859014	MH870714	–
*Gongronella chlamydospora*	CGMCC 3.16118^T^	OL678157	–	–
*Gongronella eborensis*	CCMI 1100^T^	KT809408	MN947301	–
*Gongronella eborensis*	CCMI 1101	GU244500	MN947302	–
*Gongronella fusoacuminata*	BCRC 10F0908^T^	NR_199105	NG_244365	–
*Gongronella fusoacuminata*	BCRC:10F0918	PQ496504	PQ496507	–
*Gongronella fusoacuminata*	BCRC:10F0909	PQ496503	PQ496506	–
*Gongronella guangdongensis*	CGMCC 3.15212^T^	NR_158464	MN947303	–
*Gongronella guangdongensis*	LC1994	KC462740	MN947304	–
*Gongronella hydei*	KUMCC 18.0198^T^	NR_171964	MT907273	–
*Gongronella hydei*	BRIP 74937a	OR272184	OR272185	–
*Gongronella hydei*	KUMCC_18_0204	MT152335	MT907274	–
*Gongronella inconstans*	CGMCC 3.27029^T^	PP195849	PP195950	PP933941
*Gongronella inconstans*	SAUCC 4113-3	PP195850	PP195951	PP933942
*Gongronella irregularis*	URM9013^T^	PP923717	PP923718	–
*Gongronella koreana*	EML-TS2Bp^T^	KP636529	KP636530	KP636527
*Gongronella koreana*	EML-TS2Bp-2	KP835545	KP835542	KP835543
*Gongronella lacrispora*	ATCC 24412^T^	GU244498	JN206609	–
***Gongronella longapophysata***	**URM 9240^T^**	**PZ234083**	**PZ227112**	**PZ227666**
***Gongronella longapophysata***	**URM 9241**	**PZ234084**	**PZ227113**	**PZ227667**
*Gongronella multiramosa*	CGMCC 3.26216^T^	OR733546	OR733611	PP933937
*Gongronella multiramosa*	SAUCC 4056-4	OR733545	OR733610	–
*Gongronella multispora*	CGMCC 3.16119^T^	OL678158	–	–
*Gongronella namwonensis*	CNUFC WW2-12^T^	NR_175640	MN658482	–
*Gongronella namwonensis*	XY08131	OL620098	–	–
*Gongronella oleae*	CGMCC 3.26217^T^	OR742078	OR733608	PP933945
*Gongronella oleae*	SAUCC 4164-2	OR742079	OR733609	PP933946
*Gongronella orasabula*	JMRC SF 012180^T^	NR_148087	KT936263	KT936265
*Gongronella orasabula*	EML-QF12-2	KT936270	KT936264	–
*Gongronella pamphilae*	BRIP 74936a^T^	OR271909	OR259050	–
*Gongronella pamphilae*	CGMCC 3.27027	PP195845	PP195946	PP933935
*Gongronella pamphilae*	SAUCC 4031-2	PP195846	PP195947	PP933936
*Gongronella pedratalhadensis*	URM 8182^T^	MN912512	MN912508	–
*Gongronella pingtangensis*	CGMCC 3.27032^T^	PP195855	PP195956	PP933949
*Gongronella pingtangensis*	SAUCC 5676-4	PP195856	PP195957	PP933950
*Gongronella qichaensis*	CGMCC 3.26218^T^	OR733544	OR733607	–
*Gongronella qichaensis*	SAUCC 4137-3	OR733543	OR733606	–
*Gongronella reniformis*	CGMCC 3.27030^T^	PP195851	PP195952	PP933943
*Gongronella reniformis*	SAUCC 4142-5	PP195852	PP195953	PP933944
*Gongronella sichuanensis*	GZUIFR-H25.4.1^T^	MK813373	MK813855	MK820625
*Gongronella sichuanensis*	GZUIFR-H25.4.2	MK813374	MK813856	MK820626
***Gongronella verticillata***	**URM 9238^T^**	**PZ234085**	**PZ227114**	–
***Gongronella verticillata***	**URM 9239**	**PZ234086**	**PZ227115**	–
*Gongronella zunyiensis*	GZUIFR-FX25.1^T^	MN453856	MN453853	–
*Gongronella zunyiensis*	GZUIFR-FX25.2	MN453857	MN453854	–
*Cunninghamella blakesleeana*	CBS 133.27 ^T^	NR_119974	MH866397	KJ156479
*Cunninghamella antarctica*	CBS 545.75^T^	JN205893	JN206597	KJ156492

## Data Availability

The sequences presented in this study are openly available in https://www.ncbi.nlm.nih.gov/ (see Table 1 for the accession numbers). The alignments and phylogenetic tree files are available in FigShare (https://doi.org/10.6084/m9.figshare.31438165). All new taxa were registered in the Mycobank database (www.mycobank.org). This research was registered in the Brazilian SisGen system (A77E17B).

## References

[1] Ribeiro J.F., Walter B.M.T., Sano S.M., Almeida S.P. (1998). Fitofisionomias do bioma Cerrado. Cerrado: Ambiente e Flora.

[2] Santos F.J.M. (2025). Checklist of the herpetofauna in an area of the Cerrado Biome, Central Brazil, under strong mining pressure. J. Wildl. Biodivers..

[3] De Jesus D.L.V., Aranda R. (2025). Hydromorfological soils drives ant (Hymenoptera: Formicidae) communities in Brazilian wet savanna. Wetl. Ecol. Manag..

[4] Wijayawardene N.N., Hyde K.D., Mikhailov K.V., Goto B.T., Santiago A.L.C.M.A., Tokarev Y.S., Elshahed M.S., Madrid H., Pires-Zottarelli C.L.A., Pawłowska J. (2025). Families of non-Dikarya fungi. Mycosphere.

[5] Flora e Funga do Brasil. Jardim Botânico do Rio de Janeiro. http://floradobrasil.jbrj.gov.br/reflora/listaBrasil/PrincipalUC/PrincipalUC.do;jsessionid=D8E2833E5478AF52DD9209A15B77387F#CondicaoTaxonCP.

[6] De Souza J.I., Marano A.V., Pires-Zottarelli C.L.A., Chambergo F.S., Harakava R. (2014). A new species of *Backusella* (Mucorales) from a Cerrado reserve in Southeast Brazil. Mycol. Prog..

[7] De Souza J.I., Pires-Zottarelli C.L.A., Santos J.F., Costa J.P., Harakava R. (2012). *Isomucor* (Mucoromycotina): A new genus from a Cerrado reserve in state of São Paulo, Brazil. Mycologia.

[8] Cabral L., Sauer S., Shankland A. (2025). Introduction: Reclaiming the Cerrado—A Territorial Account of a Disputed Frontier. IDS Bull..

[9] Benny G.L. (2005). Zygomycetes. http://zygomycetes.org/index.php?id=27.

[10] Voigt K. (2012). Syllabus of Plant Families.

[11] Naumov N.A. (1935). Opredelitel Mukorovych (Mucorales).

[12] Pidoplichko N.M., Mil’ko A.A. (1971). Atlas Mukoral’vykh Gribov.

[13] Mil’ko A.A. (1974). Opredelitel’mukoral’nykh Gribov.

[14] Benjamin R.K. (1959). The merosporangiferous Mucorales. Aliso.

[15] Hesseltine C.W. (1955). Genera of Mucorales with notes on their synonymy. Mycologia.

[16] Hesseltine C.W., Ellis J.J., Ainsworth G.C., Sparrow F.K., Sussman A.S. (1973). Mucorales. The Fungi: An Advanced Treatise. A Taxonomic Review with Keys: Basidiomycetes and Lower Fungi.

[17] Benny G.L., Humber R.A., Morton J.B., McLaughlin D.G., McLaughlin E.G., Lemke P.A. (2001). Zygomycota: Zygomycetes. The Mycota. Systematics and Evolution. Part A.

[18] Voigt K., Wöstemeyer J. (2001). Phylogeny and origin of 82 Zygomycetes from all 54 genera of the Mucorales and Mortierellales based on combined analysis of actin and translation elongation factor EF-1α genes. Gene.

[19] Cordeiro T.R.L., Nguyen T.T.T., Lima D.X., da Silva S.B.G., de Lima C.L.F., Leitão J.D.A., Gurgel L.M.S., Lee H.B., Santiago A.L.C.M.d.A. (2020). Two new species of the industrially relevant genus *Absidia* (Mucorales) from soil of the Brazilian Atlantic Forest. Acta Bot. Bras..

[20] Nguyen T.T.T., Santiago A.L.C.M.d.A., Hallsworth J.E., Cordeiro T.R.L., Voigt K., Crous P.M., Júnior M.A.M., Elsztein C., Lee H.B. (2024). New Mucorales from opposite ends of the world. Stud. Mycol..

[21] De Freitas L.W.S., de Oliveira R.J.V., Cordeiro T.R.L., Nguyen T.T.T., Lim H.J., Lee H.B., Santiago A.L.C.M.d.A. (2021). *Gongronella pedratalhadensis*, a new species of Mucorales (Mucoromycota) isolated From the Brazilian. Sydowia.

[22] Hoffmann K., Discher S., Voigt K. (2007). Revision of the genus *Absidia* (Mucorales, Zygomycetes) based on physiological, phylogenetic, and morphological characters; thermotolerant *Absidia* spp. form a coherent group, Mycocladiaceae fam. nov. Mycol. Res..

[23] Stencel B.M., Zień M., Jaworska M.M. (2025). The energy of activation of intracellular and extracellular chitin deacetylase. Prog. Chem. Appl. Chitin Its Deriv..

[24] Krawczyk-Łebek A., Żarowska B., Janeczko T., Kostrzewa-Susłow E. (2025). Antimicrobial activity of new glycoside derivatives of chloroflavones obtained by fungal biotransformation. Sci. Rep..

[25] Guiraud P., Villemain D., Kadri M., Bordjiba O., Steiman R. (2003). Biodegradation capability of *Absidia fusca* Linnemann towards environmental pollutants. Chemosphere.

[26] Albert Q., Leleyter L., Lemoine M., Heutte N., Rioult J.-P., Sage L., Garon D. (2018). Comparison of tolerance and biosorption of three trace metals (Cd, Cu, Pb) by the soil fungus *Absidia cylindrospora*. Chemosphere.

[27] (2026). Sepcies Fungorum. https://www.speciesfungorum.org/.

[28] Crous P.W., Wingfield M.J., Chooi Y.-H., Gilchrist C.L.M., Lacey E., Pitt J.I., Roets F., Swart W.J., Cano-Lira J.F., Valenzuela-Lopez N. (2020). Fungal Planet description sheets: 1042-1111. Persoonia.

[29] Wang Y.-X., Zhao H., Ding Z.-Y., Ji X.-Y., Zhang Z.-X., Wang S., Zhang X.-G., Liu X.-Y. (2023). Three new species of *Gongronella* (Cunninghamellaceae, Mucorales) from soil in Hainan, China based on morphology and molecular phylogeny. J. Fungi.

[30] Wang Y.-X., Zhao H., Jiang Y., Liu X.-Y., Tao M.F., Liu X.-Y. (2024). Unveiling species diversity within early-diverging fungi from China III: Six new species and a new record of *Gongronella* (Cunninghamellaceae, Mucoromycota). MycoKeys.

[31] Braga H.F., Baffi M.A., Alves P.H.F. (2023). Farinha do caroço de abacate como substrato alternativo para produção de pectinases por *Gongronella butleri*. J. Biotechnol. Biodivers..

[32] Maw T., Tan T.K., Khor E., Wong S.M. (2002). Complete cDNA sequence of chitin deacetylase from *Gongronella butleri* and its phylogenetic analysis revealed clusters corresponding to taxonomic classification of fungi. J. Biosci. Bioeng..

[33] Cavalheiro G.F., Sanguine I.S., Santos F.R.S., Costa A.C., Fernandes M., Paz M.F., Fonseca G.G., Leite R.S.R. (2017). Catalytic properties of amylolytic enzymes produced by *Gongronella butleri* using agroindustrial residues on solid-state fermentation. Biomed. Res. Int..

[34] Santos F., Garcia N.F.L., da Paz M.F., Fonseca G.G., Leite R.S.R. (2016). Production and characterization of β-glucosidase from *Gongronella butleri* by solid-state fermentation. Afr. J. Biotechnol..

[35] Peera M., Tayo-Ajimoko A., Liao F. (2024). An alternative to conventional antibiotics—The antimicrobial properties of deacetylated chitin extracted from *Gongronella butleri*: A research protocol. URNCST J..

[36] Veloso H.P., Rangel-Filho A.L.R., Lima J.C.A. (1991). Classificação da Vegetação Brasileira, Adaptada a um Sistema Universal.

[37] Martins L.P., Junior E.C.A., Martins A.R.P., Colins M.S., Almeida G.C.F., Azevedo G.G. (2017). Butterflies of Amazon and Cerrado remnants of Maranhão, Northeast Brazil. Biota Neotrop..

[38] Benny G.L. (2008). The methods used by Dr. R.K. Benjamin, and other Mycologists to isolate Zygomycetes. Aliso.

[39] Cordeiro T.R.L., Walther G., Lee H.B., Nguyen T.T.T., de Souza C.A.F., Lima D.X., de Oliveira R.J.V., Góes-Neto A., Tomé L.M.R., Kurzai O. (2023). A polyphasic approach to the taxonomy of *Backusella* reveals two new species. Mycol. Prog..

[40] Kornerup A., Wanscher J.H. (1978). Methuen Handbook of Colour.

[41] Zhao H., Nie Y., Zong T.K., Wang Y.J., Wang M., Dai Y.C., Liu X.Y. (2022). Species diversity and ecological habitat of *Absidia* (Cunninghamellaceae, Mucorales) with emphasis on five new species from forest and grassland soil in China. J. Fungi..

[42] Zhao H., Nie Y., Zong T.-K., Wang K., Lv M.-L., Cui Y.-J., Tohtirjap A., Chen J.-J., Zhao C.-L., Wu F. (2023). Species diversity, updated classification and divergence times of the phylum Mucoromycota. Fungal Divers..

[43] Ding Z.Y., Ji X.Y., Tao M.F., Liu W.X., Jiang Y., Zhao H., Meng Z., Liu X.Y. (2025). Unveiling species diversity within early-diverging fungi from China IV: Four new species of *Absidia* (Cunninghamellaceae, Mucoromycota). MycoKeys.

[44] Ji X.-Y., Ding Z.-Y., Nie Y., Zhao H., Wang S., Huang B., Liu X.-Y. (2025). Unveiling species diversity within early-diverging fungi from China V: Five new species of *Absidia* (Cunninghamellaceae, Mucoromycota). MycoKeys.

[45] Tao M.-F., Ding Z.-Y., Wang Y.-X., Zhang Z.-X., Zhao H., Meng Z., Liu X.-Y. (2024). Unveiling species diversity within early-diverging fungi from China II: Three new species of *Absidia* (Cunninghamellaceae, Mucoromycota) from Hainan Province. MycoKeys.

[46] Zhao H., Nie Y., Zong T., Dai Y., Liu X. (2022). Three New Species of *Absidia* (Mucoromycota) from China Based on Phylogeny, Morphology and Physiology. Diversity.

[47] Zong T.K., Zhao H., Liu X.L., Ren L.Y., Zhao C.L., Liu X.Y. (2021). Taxonomy and phylogeny of four new species in *Absidia* (Cunninghamellaceae, Mucorales) from China. Front. Microbiol..

[48] Urquhart A.S., Idnurm A. (2021). *Absidia healeyae*: A new species of *Absidia* (Mucorales) isolated from Victoria, Australia. Mycoscience.

[49] Luo C., Chen F., Phookamsak R., Sun J., Jiang H. (2024). Polyphasic taxonomic study of *Absidia menglianensis* sp. nov. (Cunninghamellaceae, Mucorales) isolated from an avocado plantation in Yunnan, China. Stud. Fungi.

[50] Zhao H., Zhu J., Zong T.K., Liu X.L., Ren L.Y., Lin Q., Qiao M., Nie Y., Zhang Z.D., Liu X.Y. (2021). Two new species in the family Cunninghamellaceae from China. Mycobiology.

[51] Crous P.W., Luangsa-Ard J.J., Wingfield M.J., Carnegie A.J., Hernández-Restrepo M., Lombard L., Roux J., Barreto R.W., Baseia I.G., Cano-Lira J.F. (2018). Fungal Planet description sheets: 785-867. Persoonia.

[52] Htet Y.M., Yasanthika W.E., Hurdeal V.G., de Farias A.R.G. (2024). *Absidia thailandica* sp. nov., an addition to the diversity of soil fungi from Thailand. Phytotaxa.

[53] De Freitas L.W.S., Cruz M.O., Nguyen T.T.T., Lee H.B., Santos F.R.S., Santiago A.L.C.M.A. (2023). *Absidia variicolumellata*, sp. nov., a new mucoralean fungus isolated from Atlantic Forest in Bahia state (Brazil). Sydowia.

[54] White T.J., Bruns T., Lee S., Taylor J., Innis M.A., Gelfand D.H., Sninsky J.J., White T.J. (1990). Amplification and direct sequencing of fungal ribosomal RNA genes for phylogenetics. PCR Protocols: A Guide to Methods and Applications.

[55] Vilgalys R., Hester M. (1990). Rapid genetic identification and mapping of enzymatically amplified ribosomal DNA from several *Cryptococcus* species. J. Bacteriol..

[56] Rehner S.A., Samuels G.J. (1994). Taxonomy and phylogeny of *Gliocladium* analysed from nuclear large subunit ribosomal DNA sequences. Mycol. Res..

[57] Katoh K., Standley D.M. (2013). MAFFT multiple sequence alignment software version 7: Improvements in performance and usability. Mol. Biol. Evol..

[58] Kumar S., Stecher G., Tamura K. (2016). MEGA7: Molecular evolutionary genetics analysis version 7.0 for bigger datasets. Mol. Biol. Evol..

[59] Maddison W.P., Maddison D.R. (2017). Mesquite: A Modular System for Evolutionary Analysis, Version 3.31. http://www.mesquiteproject.org.

[60] Trifinopoulos J., Nguyen L.-T., Von Haeseler A., Minh B.Q. (2016). W-IQ-TREE: A fast online phylogenetic tool for maximum likelihood analysis. Nucleic Acids Res..

[61] Stamatakis A. (2014). RAxML Version 8: A tool for phylogenetic analysis and post-analysis of large phylogenies. Bioinformatics.

[62] Miller M.A., Pfeiffer W., Schwartz T. Creating the CIPRES science gateway for inference of large phylogenetic trees. Proceedings of the 2010 Gateway Computing Environments Workshop (GCE).

[63] Hoang D.T., Chernomor O., von Haeseler A., Minh B.Q., Vinh L.S. (2018). UFBoot2: Improving the ultrafast bootstrap approximation. Mol. Biol. Evol..

[64] Kalyaanamoorthy S., Minh B.Q., Wong T.K.F., von Haeseler A., Jermiin L.S. (2017). ModelFinder: Fast model selection for accurate phylogenetic estimates. Nat. Methods.

[65] Ronquist F., Teslenko M., van der Mark P., Ayres D.L., Darling A., Hȯhna S., Larget B., Liu L., Suchard M.A., Huelsenbeck J.P. (2012). MrBayes 3.2: Efficient bayesian phylogenetic inference and model choice across a large model space. Syst. Biol..

[66] Rambaut A. (2016). FigTree (Tree Figure Drawing Tool), version 1.4. 3 2006–2016.

[67] Tan Y.P., Bishop-Hurley S.L., Conroy J.R., Shivas R.G. (2025). Index of Australian Fungi No. 50. https://zenodo.org/records/14693768.

[68] Paine F.S. (1927). Studies on fungous flora of virgin soils. Mycologia.

[69] Hesseltine C.W., Ellis J.J. (1964). An interesting species of *Mucor*, *M. ramosissimus*. Sabouraudia.

[70] Schipper M.A.A. (1990). Notes on Mucorales—I. Observations on *Absidia*. Persoonia.

[71] Kirk P.M., Cannon P., Stalpers J., Minter D.W. (2008). Dictionary of the Fungi.

[72] Hoffmann K., Walther G., Voigt K. (2009). *Mycocladus* vs. *Lichtheimia*: A correction (Lichtheimiaceae fam. nov., Mucorales, Mucoromycotina). Mycol. Res..

[73] Hoffmann K., Voigt K. (2009). *Absidia parricida* plays a dominant role in biotrophic fusion parasitism among mucoralean fungi (Zygomycetes): *Lentamyces*, a new genus for *A. parricida* and *A. zychae*. Plant Biol..

[74] Lima D.X., Cordeiro T.R., De Souza C.A., de Oliveira R.J., Lee H.B., Souza-Motta C.M., Santiago A.L.D.A. (2020). Morphological and molecular evidence for two new species of *Absidia* from Neotropic soil. Phytotaxa.

[75] Leitaão J.D., Cordeiro T.R., Nguyen T.T., Lee H.B., Gurgel L.M., Santiago A.L.C.M.d.A. (2021). *Absidia aguabelensis* sp. nov.: A new mucoralean fungi isolated from a semiarid region in Brazil. Phytotaxa.

[76] Hesseltine C.W., Ellis J.J. (1961). Notes on Mucorales, especially *Absidia*. Mycologia.

[77] Upadhyay H.P. (1970). Soil fungi from north-east and north Brazil—VII. The genus *Gongronella*. Persoonia.

[78] Zheng R.-Y., Chen G.-Q. (2001). A monograph of *Cunninghamella*. Mycotaxon.

[79] De Lima C.L.F., Cordeiro T.R.L., dos Santos M.A.B., Leão I.F., dos Santos F.R.S., Muniz A.W., Yadav L.S., Lee H.B., Santiago A.L.C.M. (2026). The third report of Cunninghamellaceae fungi in the Brazilian Amazon forest: Two new species of *Cunninghamella* isolated from soil. Mycologia.

[80] Alastruey-Izquierdo A., Hoffmann K., de Hoog G.S., Rodriguez-Tudela J.L., Voigt K., Bibashi E., Walther G. (2010). Species recognition and clinical relevance of the zygomycetous genus *Lichtheimia* (syn. *Absidia* Pro Parte, *Mycocladus*). J. Clin. Microbiol..

[81] Wagner L., Stielow J.B., de Hoog G.S., Bensch K., Schwartze V.U., Voigt K., Alastruey-Izquierdo A., Kurzai O., Walther G. (2020). A new species concept for the clinically relevant *Mucor circinelloides* complex. Persoonia.

[82] Zheng R.-Y., Chen G.-Q., Huang H., Liu X.-Y. (2007). A monograph of *Rhizopus*. Sydowia.

[83] Ariyawansa H.A., Hyde K.D., Jayasiri S.C., Buyck B., Chethana K.W.T., Dai D.Q., Dai Y.C., Daranagama D.A., Jayawardena R.S., Lücking R. (2015). Fungal diversity notes 111–252—Taxonomic and phylogenetic contributions to fungal taxa. Fungal Divers..

[84] Crous P.W., Cowan D.A., Maggs-Kölling G., Yilmaz N., Thangavel R., Wingfield M.J., Noordeloos M.E., Dima B., Brandrud T.E., Jansen G.M. (2021). Fungal Planet description sheets: 1182–1283. Persoonia.

[85] Tibpromma S., Hyde K.D., Jeewon R., Maharachchikumbura S.S.N., Liu J.K., Bhat D.J., Jones E.B.G., Mckenzie E.H.C., Camporesi E., Bulgakov T.S. (2017). Fungal diversity notes 491–602: Taxonomic and phylogenetic contributions to fungal taxa. Fungal Divers..

[86] Crous P.W., Wingfield M.J., Jurjević Ž., Balashov S., Osieck E.R., Marin-Felix Y., Luangsa-ard J.J., Mejía L.C., Cappelli A., Parra L.A. (2024). Fungal Planet description sheets: 1697–1780. Fungal Syst. Evol..

